# Overexpression of Endoglin Modulates TGF-β1-Signalling Pathways in a Novel Immortalized Mouse Hepatic Stellate Cell Line

**DOI:** 10.1371/journal.pone.0056116

**Published:** 2013-02-20

**Authors:** Steffen K. Meurer, Muhammad Alsamman, Hacer Sahin, Hermann E. Wasmuth, Tatiana Kisseleva, David A. Brenner, Christian Trautwein, Ralf Weiskirchen, David Scholten

**Affiliations:** 1 Institute of Clinical Chemistry and Pathobiochemistry, RWTH University Hospital Aachen, Aachen, Germany; 2 Department of Internal Medicine III, RWTH University Hospital Aachen, Aachen, Germany; 3 Department of Medicine, University of California San Diego, La Jolla, California, United States of America; Cleveland Clinic Lerner Research Institute, United States of America

## Abstract

Hepatic stellate cells (HSCs) play a major role in the pathogenesis of liver fibrosis. Working on primary HSCs requires difficult isolation procedures; therefore we have generated and here characterize a mouse hepatic stellate cell line expressing GFP under control of the collagen 1(I) promoter/enhancer. These cells are responsive to pro-fibrogenic stimuIi, such as PDGF or TGF-β1, and are able to activate intracellular signalling pathways including Smads and MAP kinases. Nevertheless, due to the basal level of activation, TGF-β1 did not significantly induce GFP expression contrasting the TGF-β1 regulated endogenous collagen I expression. We could demonstrate that the accessory TGF-β-receptor endoglin, which is endogenously expressed at very low levels, has a differential effect on signalling of these cells when transiently overexpressed. In the presence of endoglin activation of Smad1/5/8 was drastically enhanced. Moreover, the phosphorylation of ERK1/2 was increased, and the expression of vimentin, α-smooth muscle actin and connective tissue growth factor was upregulated. Endoglin induced a slight increase in expression of the inhibitor of differentiation-2 while the amount of endogenous collagen type I was reduced. Therefore, this profibrogenic cell line with hepatic stellate cell origin is not only a promising novel experimental tool, which can be used *in vivo* for cell tracing experiments. Furthermore it allows investigating the impact of various regulatory proteins (e.g. endoglin) on profibrogenic signal transduction, differentiation and hepatic stellate cell biology.

## Introduction

In response to liver injury, hepatic stellate cells (HSCs) transdifferentiate from a quiescent vitamin A storing phenotype into activated myofibroblast-like cells (MFBs) [1]. HSCs modulate inflammation and extracellular matrix (ECM) deposition and are the major source for collagen formation in injured livers [2]. Due to this pivotal role in liver pathology, HSCs have been in the focus of scientific research for many years. Ever since, the overall plasticity of these cells has been a scientific challenge. Nevertheless, working with primary HSCs requires a complex isolation and sorting process, which usually shows low yields, is time consuming, expensive and allows only limited numbers of experiments [3]. Therefore, several immortalized HSCs cell lines have been used for *in vitro* experiments for many years. Unfortunately all of these cell lines represent cells with a myofibroblast-like phenotype, a strongly reduced plasticity and considerable different protein expression patterns compared to primary HSCs/MFBs. [4]. Due to their developmental origin from the neurogenic crest HSCs are the only liver cells, which express glial fibrilliary acidic protein (GFAP), neuropilin, synaptophysin and p75-receptor [5], [6]. Upon activation and transdifferentiation to MFBs, HSCs lose their vitamin A droplets and upregulate expression of mesenchymal markers such as α-smooth muscle actin (α-SMA), desmin, vimentin and fibronectin [7].

TGF-β1 is a major profibrogenic cytokine. It acts through multiple mechanisms, including direct activation of HSCs and stimulation of ECM production as well as prompting the synthesis of tissue inhibitors of matrix metalloproteases (TIMPs), thereby inhibiting ECM degradation [8]. Collagen type I is a key matrix component regulated by TGF-β1 in fibrosis [2], [9]. Furthermore, many physiological and pathological processes such as proliferation, cellular differentiation and apoptosis are regulated by the TGF-β ligand family [10].

There are three different TGF-β isoforms (TGF-β1, -β2, -β3) expressed, which bind to a heterooligomeric receptor complex located in the cell membrane. For TGF-β1 this complex comprises dimers of the type I receptor (TβRI) ALK5 and the type II receptor (TβRII). Binding of TGF-β1 to TβRII leads to co-assembly with ALK5, which transfers the signal to the intracellular compartment via phosphorylation of Smad proteins, i.e. Smad2 and Smad3 [11]. These in turn translocate to the nucleus, where they interact with other transcriptional co-activators or co-repressors to regulate gene expression [12]. In several cell types, including HSCs, TGF-β1 engages an alternative type I receptor, i.e. ALK1. In contrast to ALK5, ALK1 acts upon phosphorylation of Smad1, Smad5 and Smad8 to regulate a different subset of genes [13], [14]. β-glycan and endoglin are the two type III TGF-β receptors. Endoglin (CD105) is a disulfide-linked, homodimeric transmembrane glycoprotein [15], which is highly expressed on proliferating vascular endothelial cells [16], fibroblasts [17], macrophages [18], vascular smooth muscle cells and HSCs [19]. Endoglin binds different ligands of the TGF-β-superfamily in the presence of TβRI and TβRII [20]. As an auxiliary TGF-β co-receptor it modulates the balance between TGF-β1-ALK1 and TGF-β1-ALK5 signalling pathways [21].

Recently, endoglin expression was linked to fibrotic diseases. It is expressed on human mesangial cells and modulates ECM synthesis [22] as well as it impacts on fibroblast function [19]. Rat HSCs and MFBs express high amounts of endoglin; thereby tuning the two different interconnected signalling pathways of TGF-β [20], [23]. We recently could show that endoglin modulates TGF-β1-signalling and differentiation of CFSC-2G cells, an immortalized cell line of rat HSC origin [24]. Moreover, patients with hepatitis C infection [23] or liver cirrhosis show high levels of shedded, soluble endoglin [25].

We here generate and characterize a novel immortalized murine HSC cell line that carries the GFP transgene under the control of the collagen α1(I) promoter/enhancer and demonstrate that this cell line is a promising tool which can be used to investigate special issues of profibrogenic signalling. In this cell line endoglin modulates TGF-β1 Smad/non-Smad signalling pathways resulting in different fibrogenic properties of these immortalized HSCs.

## Materials and Methods

### Isolation and Culturing of Primary Hepatic Stellate Cells

Primary HSCs from normal C57BL/6 and Col-GFP transgenic mice as well as from Sprague Dawley rats were isolated using Nycodenz gradient centrifugation and cultured as described before [26], [27], [28]. Source and culture conditions of GRX cells, CFSC-2G, HSC Col-GFP, HSC Sv40/mTert, HepG2, and COS-7 cells are listed in Table S1. Dulbecco’s modified Eagle’s medium (Lonza, Walkersville, MD, USA), fetal calf serum (FCS, Perbio Science, Cramlington, UK), 4 mM L-Glutamine, 100 IU/ml penicillin, and 100 µg/ml streptomycin (all from Cambrex, Verviers, Belgium) and non essential aminoacids (for CFSC, Lonza, Walkersville, MD, USA) were taken to prepare final media.

### Establishment, Generation and Culturing of a Col-GFP Immortalized Hepatic Stellate Cell Line

A lentivirus vector containing the SV40 large T antigen (kind gift from Dr. Jean Y. J. Wang, University of California, San Diego, CA) and a hygromycin resistance gene was generated in the 293 Phoenix eco cell line (Invitrogen, Life Technologies, Darmstadt, Germany). The purified vector, AL-118 Polybrene (Sigma, Taufkirchen, Germany) and hygromycin (Sigma) was added to the primary cultures of Col-GFP cells. Hygromycin resistant colonies were identified and single cell clones from Hygromycin^+^ GFP^+^ cells were generated.

### Plasmids

Both the luciferase reporter construct (CAGA)_12_-MLP-Luc and the overexpression construct of constitutively active human ALK5 (ca-ALK5) were kind gifts of Dr. Peter ten Dijke, Leiden University Medical Center, The Netherlands. The expression vector for rat endoglin (L-form, pcDNA-endoglin) have been described before [29], [30]. The expression vector for mouse endoglin (IRAVp968G0448D6) was purchased from imaGenes GmbH (Berlin, Germany).

### Transient Transfection Experiments

COS-7 and murine HSCs were cultured in growth medium (GM, see Table S1) till 80% confluent. For transient transfection, cell lines were plated into 6-well dishes at a density of 2.5–3×10^5^ per well. COS-7 cells were transfected with 2 µg of DNA and 6 µl Mirus transfection reagent (Mirus, VWR International GmbH, Darmstadt, Germany). Murine HSCs were transfected with 2 µg DNA and 4 µl Lipofectamine 2000 reagent (Invitrogen). After 24 hrs, medium was renewed and cells were either used for stimulation experiments or proteins were extracted in lysis buffer [50 mmol/l Tris/HCl (pH 7.2), 250 mmol/l NaCl, 2% (v/v) NP-40, 0.1% (w/v) SDS, 0.5% (w/v) sodium deoxycholate, 2.5 mM EDTA] containing the Complete™-cocktail of proteinase inhibitors (Roche, Mannheim, Germany) and of the phosphatase inhibitor cocktail set II (Sigma) at a dilution of 1∶100.

### Stimulation and Inhibition Experiments

Col-GFP cells were cultured in growth medium containing 10% (v/v) FCS. Next, medium was changed to serum-free DMEM and cells were incubated with 100 or 250 ng CXCL9/ml (R&D Systems, Wiesbaden, Germany), 100 or 200 ng/ml PDGF (Sigma) or different concentrations (0.5, 1, 5, 10 and 25 mg/ml) Acetylcysteine (Hexal AG, Holzkirchen, Germany) for 24 hrs. Thereafter, protein or mRNA was isolated as described previously. For stimulation with recombinant TGF-β1 (0.1/1.0 ng/ml), BMP-2, BMP-7, PDGF-BB (25 ng/ml each) or EGF (50/100 ng/ml) (all obtained from R&D Systems), the serum was reduced to 0.5% FCS for 16 hrs and further lowered to 0.2% during addition of indicated concentrations of respective cytokines. When indicated, the cells were pre-treated for 30 min with 5 µM SB431542 (Tocris Bioscience, BIOZOL, Eching, Germany) or 1 µM Dorsomorphin (Biomol, Hamburg, Germany). respectively. After indicated time intervals, the cellular proteins were extracted using RIPA lysis buffer. Proteins in the lysates were quantified using the DC assay reagent (Bio-Rad, Munich, Germany) and analyzed by Western blots.

### Immunofluorescence Analysis

Formalin-fixed frozen liver tissues from CCl_4_-treated collagen α1(I)-GFP mice were analyzed for GFP expression without further staining using an Olympus IX71 fluorescence microscope (Olympus, Melville, NY, USA).

### Immunocytochemical Analysis

For immunofluorescence staining of Col-GFP transgenic cells, cells were fixed in 4% Paraformaldehyde. After blocking with 0,2% BSA for 30 min, cells were stained using specific antibodies against α-SMA, GFAP, Synaptophysin or the appropriate isotype control, followed by secondary Alexa Fluor antibody and nuclei co-staining with 4,6-diamidino-2-phenylindole. All antibodies and dilutions used in this analysis are given in Table S2. For GFP expression analysis, Col-GFP cells were plated in DMEM medium containing 10% FCS medium overnight in 96 well cell culture dishes at a density between 7 and 8×10^3^ cells/well. Cells were kept in serum free DMEM medium for 24 hrs prior to stimulation. Analysis of GFP expression was done with an FLx800 Fluorescence microplate reader (BioTek, Bad Friedrichshall, Germany) at excitation 400 nm and emission 508 nm.

### Western Blot Analysis

For Western blot analysis, cultured cells were washed in ice-cold PBS solution and extracted in RIPA lysis buffer containing proteinase and phosphatase inhibitors. Equal amounts of protein lysates were diluted under reducing conditions in NuPAGE LDS electrophoresis sample buffer (Invitrogen), heated at 75°C for 10 min and separated in 4–12% Bis-Tris gels (Invitrogen) using MOPS-SDS running buffer [50 mmol/l 3-(N-morpholino)-propane sulfonic acid, 50 mmol/l Tris-HCl (pH7.7), 3.47 mmol/l SDS, and 1.025 mmol/l EDTA] or MES-SDS running buffer [50 mmol/l 2-(N-morpholino)-ethane sulfonic acid, 50 mmol/l Tris-HCl (pH 7.3), 3.47 mmol/l SDS, and 1.025 mmol/l EDTA], respectively. Proteins were electroblotted onto nitrocellulose membranes (0.2 µm, Schleicher & Schuell, Dassel, Germany) using NuPAGE transfer buffer (Invitrogen) and equal protein loading was monitored in Ponceau S stain and by probing with a β-actin antibody. Unspecific binding sites were blocked in TBST [10 mM Tris-HCl, 150 mM NaCl, 0.1% (v/v) Tween 20, pH 7.6] containing 5% (w/v) non-fat milk powder. Primary antibodies used are listed in Table S2. Primary antibodies were visualized using horseradish-peroxidase (HRP)-conjugated secondary antibodies (Santa Cruz Biotechnology, Santa Cruz, CA, USA) using the SuperSignal West Dura Extended Duration substrate (Perbio Science).

The densitometric analysis was done using the LumiAnalyst software (version 3.1) and the Lumi-Imager system (both from Roche). Band intensities were normalised to β-actin and the intensity of the control were set to 1 and other intensities were given as fold induction.

### RNA Isolation, cDNA Synthesis and Quantitative RT-PCR Analysis

Total RNA was extracted from adherent cells using the Purelink RNA Mini Kit (Invitrogen) according to the manufacturer’s manual with on-column DNA digestion. For RT-PCR-experiments, purified samples of total RNA (1 µg each) were reverse transcribed at 42°C for 60 min using the Superscript II reverse transcriptase kit (Invitrogen) and random hexamer primers. Aliquots of first strand cDNAs were subjected to PCR in 1×PCR buffer [10 mmol/l Tris-HCl (pH 8.3), 50 mmol/l KCl, 1.5 mmol/l MgCl_2_] using 2 µM forward/reverse primers, 0.2 mM each dATP, dCTP, dGTP, dTTP, and 2.5 U of *Taq* DNA polymerase (Roche). Quantitative real time PCR (qRT-PCR) was performed on a 7300 Real-Time PCR System (Applied Biosystems, Foster City, CA, USA) using the SYBR GreenER qPCR SuperMix reagent system for ABI PRISM Instrument (Invitrogen) according to the manufacturer’s instructions. Cycle conditions and primer combinations that were used in this study are given in Table S3. The results were analyzed *via* the 2^−ΔΔCt^ method with *β-actin* as reference gene.

### Statistics

Results are presented as the mean of three independent experiments (±SEM). Statistical analyses were performed with an unpaired Student’s t-test.

## Results

### Generation of Col-GFP Immortalized Hepatic Stellate Cells

**Figure 1:**
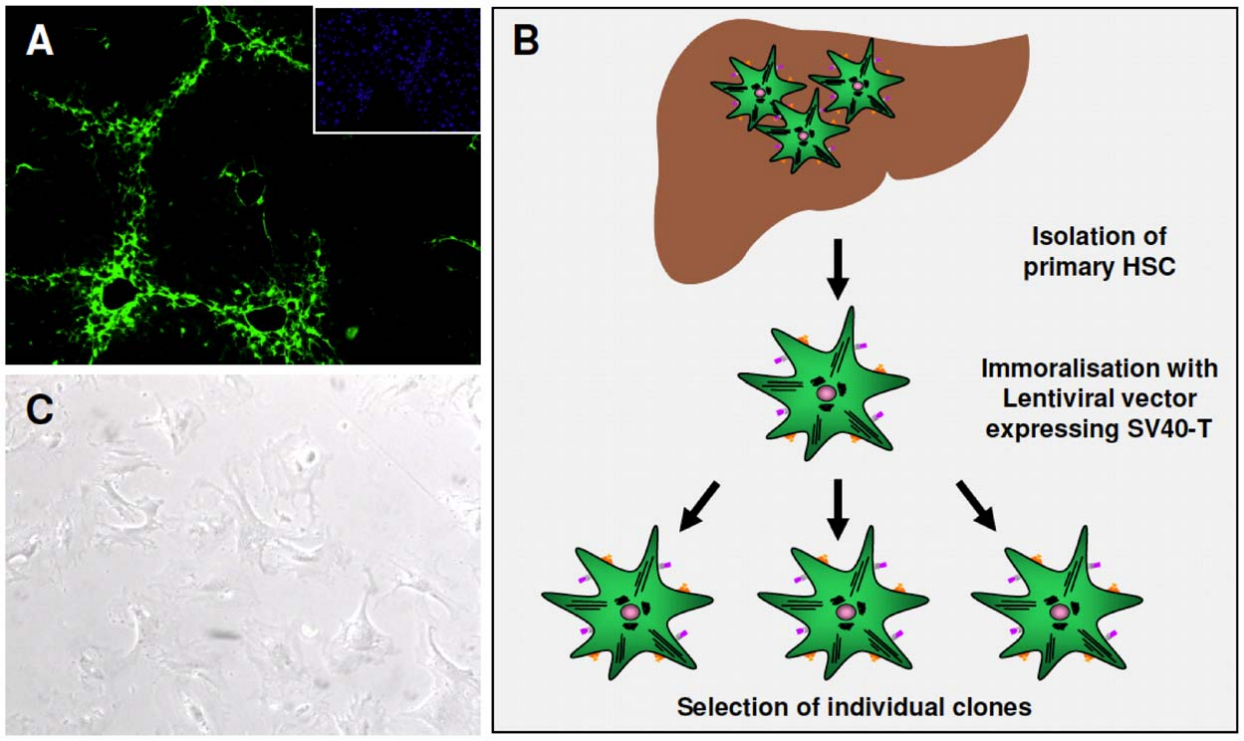
Generation of Col-GFP carrying immortalized cells.

HSCs were isolated from Col-GFP mice expressing green fluorescent protein (GFP) under control of the collagen α1(I) promoter/enhancer (Figure 1A and 1B) [27], [28]. These mice were treated with CCl_4_ for 8 weeks, the collagen promoter activity in activated HSCs is reflected by GFP expression. The inlet shows an untreated Col-GFP mouse with no collagen promoter activity. Vitamin A droplets are detectable in liver tissue from untreated mice by autofluorescence using a blue filter. In fact, HSC isolation and FACS sorting is simply based on this autofluorescence as reported before [27], [28]. This autofluorescence is decreased in liver tissue from CCl_4_ treated mice because activated HSCs lose vitamin A droplets. To generate an immortalized cell line, HSCs were infected with a lentiviral vector construct expressing SV40-large T antigen as well as a hygromycin resistance gene. Individual cell clones were isolated by selection in medium containing hygromycin (Figure 1C).

### Characterization of Immortalized Col-GFP Stellate Cells

**Figure 2:**
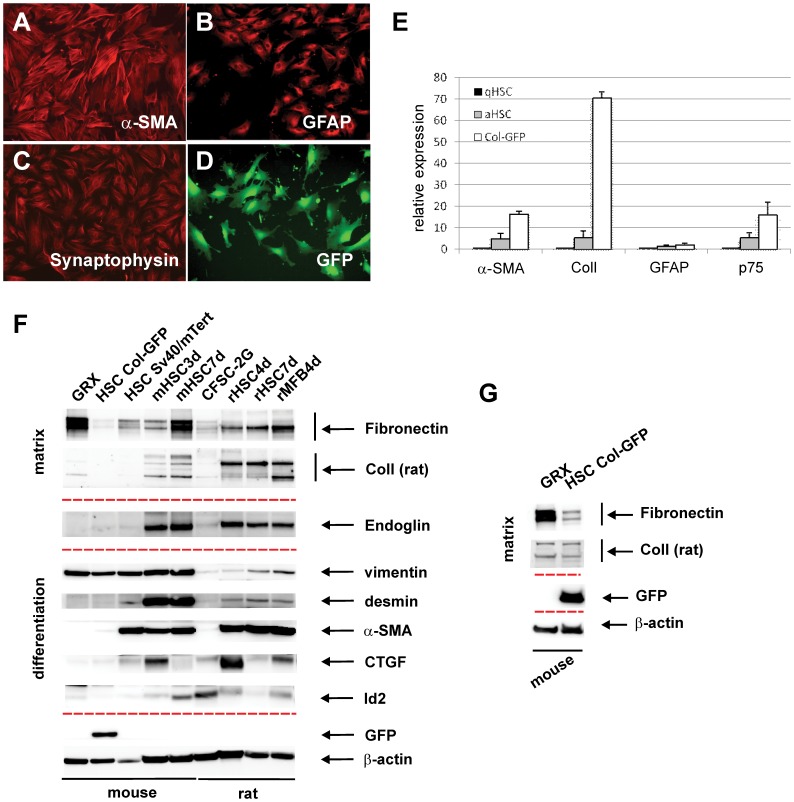
Characterization of the Col-GFP cell line. Immunofluorescence staining of cells carrying the Col-GFP reporter cassette for α-SMA (**A**), GFAP (**B**), synaptophysin (**C**). (**D**) The GFP signals were detected by UV fluorescence microscopy. (**E**) The expression of α-SMA, collagen type I, GFAP, and p75 was analyzed by quantitative PCR and compared to those obtained in quiescent primary HSC (qHSC), and activated HSC cultured for 7 days (aHSC). Results represent three independent experiments, each experiment was done in duplicates, and error bars represent SEM values. (**F**) Cells were cultured in the respective growth media and cellular proteins were extracted and analyzed by Western blot using specific antibodies to Fibronectin, ColIV, ColI, β-glycan (crossreactive with Endoglin, [20]), vimentin, desmin, α-SMA, CTGF, Id2, GFP and as a loading control β-actin, respectively. (**G**) To demonstrate expression of ColI, an image with a longer exposure time is shown (n = 3).

To characterize the isolated HSC cell line, we first analyzed the immortalized Col-GFP cells for typical markers of HSCs/MFBs. Immunofluorescent staining for α-SMA, GFAP and Synaptophysin revealed expression of these mesenchymal and neurogenic markers (Figure 2A–C). A strong expression of GFP reflects the activation of the collagen α1(I) promoter/enhancer as it is seen in activated, collagen producing HSCs (Figure 2D). Immunohistochemistry staining shows a co-expression of mesenchymal and neurogenic markers together with the activation marker collagen α1(I), a characteristic for activated hepatic stellate [31]. We concluded that the immortalized cells represent a highly pure population of activated HSCs.

Next, the properties of immortalized HSCs were compared to the primary qHSCs or in vitro activated aHSCs by RT-PCR. Compared to quiescent and activated HSCs, immortalized Col-GFP cells express 16 times higher mRNA quantities of α-SMA and p75. Moreover, the expression of collagen α1(I) was upregulated 70 times compared to quiescent HSCs, while GFAP expression showed comparable results as observed in activated primary HSCs. The high upregulation of α-SMA and collagen mRNA reflects a strong degree of activation that resembles those observed in MFBs and the ability to express GFAP again shows that the collagen α1(I) positive cells are of stellate cell origin. The high upregulation of Col-GFP in immortalized cells reflects a myofibroblast-like phenotype after several passages *in vitro*.

Western Blot analysis showed expression of fibronectin, collagen IV (not shown), and collagen I, all components of ECM typically produced by activated HSCs (Figure 2F). The mesenchymal and neurogenic markers such as vimentin and GFAP (not shown) are expressed in the liver by HSCs exclusively. In addition, desmin, the activation marker α-SMA, and the fibrosis-associated protein connective tissue growth factor (CTGF) as well as the inhibitor of differentiation-2 (Id2) are expressed. Comparison of the expression levels between cell lines and primary cells of different species reveals that analyzed markers are lower expressed in all immortalized cell lines. This effect was especially pronounced for endoglin (see also below). In summary the expression analysis revealed that immortalized Col-GFP cells express mesenchymal next to neurogenic markers and produce parts of the extracellular matrix such as fibronectin and collagen. Therefore, we conclude that these cells are MFBs with HSC origin.

### Analysis of GFP Expression

**Figure 3:**
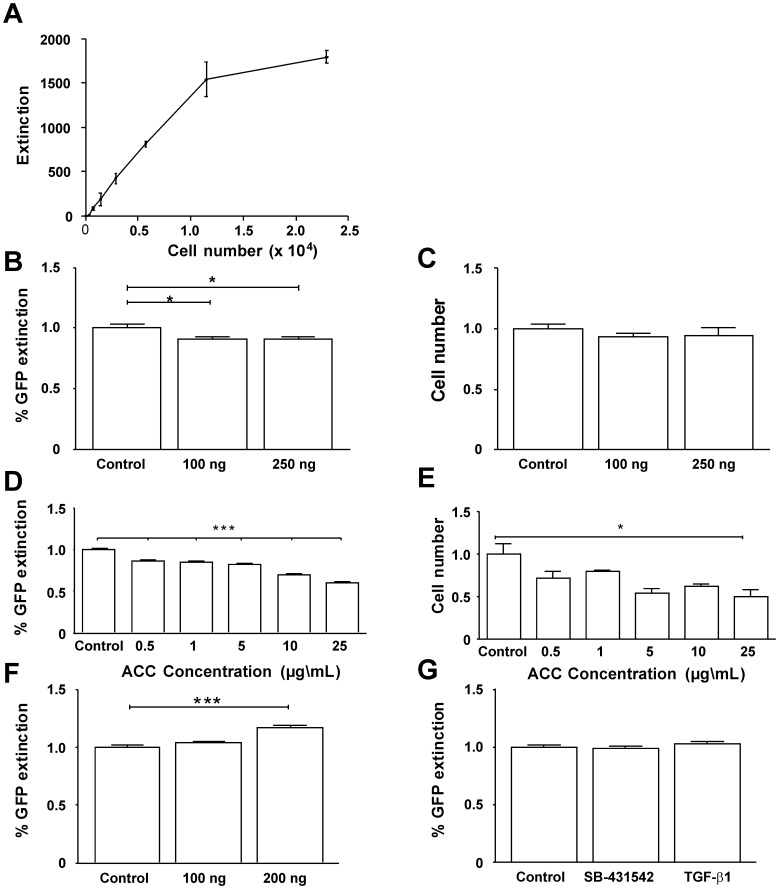
Functional characterization by fluorescence signal. (**A**) Col-GFP cells were grown to different cell densities and the fluorescence signal measured in an automated fluorescence reader. Following experiments were performed after plating cells at a density of 1.5×10^3^/well. (**B, C**) Cells were stimulated with indicated concentrations of CXCL9 for 24 hrs and the GFP content relative to untreated cells (**D**) and the cell number (**E**) was measured. (**D, E**) Cells were stimulated with indicated concentrations of ACC for 24 hrs and the GFP content relative to untreated cells (**D**) and the cell number (**E**) was measured. (**F**) Shows stimulation with indicated concentrations of PDGF-BB for 24 hrs, GFP content relative to untreated cells was measured. The increase of GFP reflects an increased cell number (not shown). Results represent three independent experiments, each experiment was done in 16 replicates, and error bars represent SEM.

To analyze GFP expression in more detail, Col-GFP cells were cultured in 96-well cell culture plates and the extinction was analyzed using an automated fluorescence reader. Figure 3A shows a linear correlation between the number of cells plated and the amount of GFP expression. Based on a linear extinction in the range of 0 to 1.5×10^4^ cells, we performed further experiments with a concentration of 5×10^3^ cells/well that place the expected GFP expression in the linear range of GFP measurement.

To analyze if the immortalized cells respond to antifibrotic stimuli, such as CXCL9 known to reduce HSC viability, Col-GFP cells were stimulated with 100 and 250 ng CXCL9 [32] for 24 hrs. We could show a significant (p<0.05) decrease of GFP extinction by about 8.8% (Figure 3B). This was due to a decreased cell number/well (Figure 3C), reflecting the previous reported anti-proliferative properties of CXCL9. Similar results were obtained by co-incubation of Col-GFP cells with Acetylcysteine (ACC) in different concentrations. ACC is known to block TGF-β1 signalling at different molecular steps including disaggregation of the biologically active TGF-β1 dimer, reduced TGF-β1 binding activity to the transforming growth factor β type III receptor (TβRIII) β-glycan, and a decomposition of endoglin representing a second accessory TβRIII receptor [30], [33]. These experiments reveal that increasing ACC concentrations result in a highly significant (p<0.001) decrease of GFP extinction by up to 41% (Figure 3D), which reflects anti-proliferative properties of ACC. In response to ACC co-incubation the cell number was reduced significantly (p<0.05) to two thirds of the untreated controls (Figure 3E). As expected treatment of Col-GFP cells with a strong profibrogenic agent such as PDGF-BB, induced proliferation (not shown) and was further linked with a highly significant (p<0.001) increase of approximately 16% GFP expression (Figure 3F). However, TGF-β1 treatment did not show any change in GFP extinction (Figure 3G), reflecting the already highly activated MFB phenotype of these immortalized cells that are known to display decreased availability of surface receptors for TGF-β [34].

### Sensitivity of Col-GFP Cells Towards Ligands Involved in Fibrosis

**Figure 4:**
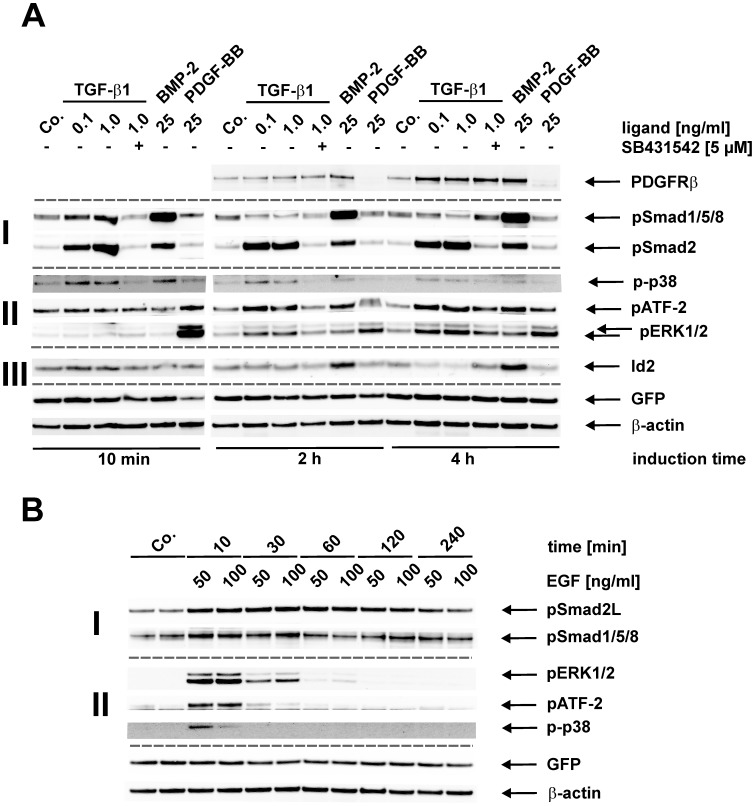
Stimulation of fibrogenic signalling in Col-GFP cells. (**A**) Cells were stimulated with indicated concentrations of TGF-β1 (0.1 ng/ml; 1.0 ng/ml), BMP-2 (25 ng/ml), PDGF-BB (25 ng/ml) or left untreated (Co.) for indicated time intervals from 10 min to 4 hrs. When indicated, the cells were treated with the ALK5 inhibitor SB431542 (5 µM). Cell extracts were prepared and cellular proteins tested for expression of PDGFRβ, phosphorylated Smad1/5/8 (pSmad1/5/8) and Smad2 (pSmad2) (I), the phosphorylated forms of p38 (p-p38) and ERK1/2 (pERK1/2), phosphorylated ATF-2 (pATF-2) (II), Id2 and GFP by Western blot. (**B**) Cells were stimulated for the indicated time intervals with EGF (50 ng/ml; 100 ng/ml). Thereafter, cell extracts were prepared and analyzed by Western blot using specific antibodies to linker phosphorylated Smad2 (pSmad2L), phosphorylated Smad1/5/8 (pSmad1/5/8) (I), the phosphorylated forms of p38 (p-p38) and ERK1/2 (pERK1/2), phosphorylated ATF-2 (pATF-2) (II) and GFP. Membranes (**A, B**) were incubated with an antibody specific for β-actin to demonstrate equal protein loading. Band intensities of the indicated proteins (**A, C**) were measured, normalized to β-actin and represented as fold induction of the unstimulated sample. Results show representative images of one of three independent experiments.

To evaluate the suitability of these cells as “model system” to analyze fibrogenic signal processing, we first treated cells with TGF-β1 in a time- and concentration-dependent manner (Figure 4, Figures S1 and S2) showing that phosphorylation of p42 is about fortyfold induced by PDGF-BB and Smad2 by TGF-β1 over tenfold (Figure S3A). In addition, the phosphorylation of the linker region in Smad2 (pS2L), Smad1/5/8, p42, pATF-2, and p38 were triggered by EGF by factors in the range of two to twenty (Figure S3B). Both Smad pathways, i.e. ALK5/Smad2/Smad3 and ALK5/Smad1/Smad5/Smad8, were instantaneously (10 min) phosphorylated in response to TGF-β1 (Figure 4A, I). In contrast to the sustained activation of Smad2 (up to 4 hrs), Smad1/5/8 activation is only transient and increases up to 1 h and is repressed starting at 2 hrs after stimulation. This activation pattern parallels the expression of Id2 (with a slight delay, compared to Smad1/5/8 repression), a direct target gene of Smad1/5/8 (Figure 4A, III). Since the ALK5 inhibitor SB431542 abrogates activation of both Smad pathways, ALK5 is an essential component for both of these responses (Figure 4A, I, II).

In contrast to TGF-β1, BMP-2 causes a strong and persistent activation of Smad1/5/8, its target gene Id2, and a weak prolonged activation of Smad2, which is comparable to the effect obtained after treatment with 0.1 ng/ml TGF-β1. PDGF-BB alone had no effect on Smad activation.

With respect to MAP-kinases, TGF-β1 induces a faint but rapid activation of p38 (starting after 10 min stimulation) and a delayed activation of ERK1/2 (starting 1 h after stimulation), which parallels activation of ATF-2 (Figure 4A, II). Again, these responses rely on ALK5 activity, because SB431542 is able to block the activation of both MAPK. BMP-2 causes rapid activation of p38, similar to TGF-β1, delayed activation of ATF-2 but has apparently no effect on ERK1/2. On the other hand, PDGF-BB transiently activates ERK1/2, ATF-2, and expression of GFP but has obviously no effect on p38.

EGF, another critical agonist in liver fibrogenesis, leads to a rapid and transient activation of p38, ERK1/2 and the substrate ATF-2 (Figure 4B, II). Furthermore, a convergence on the Smad pathways could be shown, since EGF causes a quick and transient phosphorylation of the linker region of Smad2 (comparable to PDGF-BB, data not shown) and a faint activation of the C-terminal region of Smad1/5/8 (Figure 4B, I).

### TGF-β1 Signalling in CoI-GFP Cells

Using short (10 min) and intermediate time points (up to 4 h) for stimulation, we defined the time limits of signalling (see above) as well as critical regulatory points (switch between induction and reduction of Id expression).

**Figure 5:**
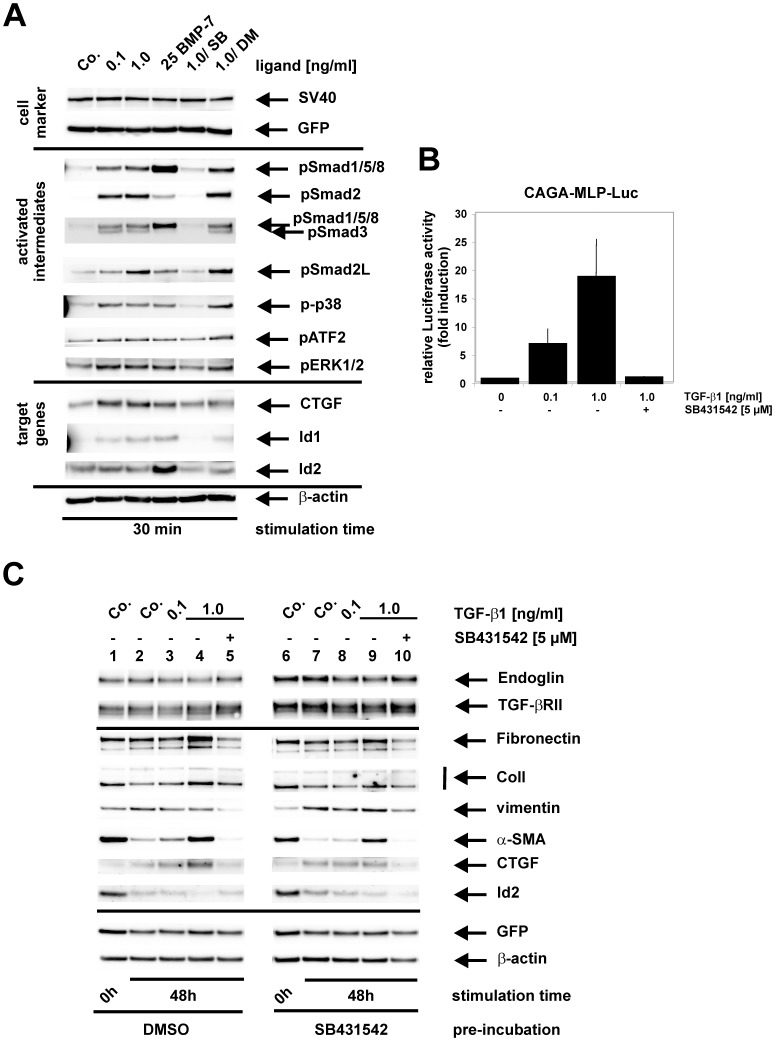
Detailed analysis of TGF-β1 mediated short and long-term responses. (**A**) Cells were stimulated for 30 min with TGF-β1 (0.1 ng/ml; 1.0 ng/ml), BMP-7 (25 ng/ml) or left untreated (Co.). When indicated, the cells were stimulated in the presence of SB431542 (SB, 5 µM) or Dorsomorphin (DM, 1 µM). Thereafter, protein extracts were analyzed by Western blot using specific antibodies to phosphorylated Smad1/5/8 (pSmad1/5/8), C-terminally-phosphorylated Smad2 (pSmad2), phosphorylated Smad3 (pSmad3), the phosphorylated forms of p38 (p-p38) and ERK1/2 (pERK1/2), CTGF, Id1, SV40-large T antigen (SV40), and GFP. (**B**) Cells were transiently transfected with the pSmad3-responsive luciferase reporter (CAGA)_12_-MLP-Luc. Thereafter, cells were stimulated with (0.1 ng/ml; 1.0 ng/ml) or without (0) TGF-β1 for 6 hrs in the presence or absence of SB431542 (5 µM). Cells were lysed and the luciferase activity determined, normalized to the protein content of the corresponding sample and expressed as fold induction to unstimulated control samples. (**C**) Cells were stimulated for 48 hrs with TGF-β1 (0.1 ng/ml; 1.0 ng/ml) or left untreated (Co.) and when indicated pre-incubated with SB431542 (SB, 5 µM) during starvation (∼16 hrs). Thereafter, a first sample was taken to monitor protein expression before stimulation (Co., 0 h). After the stimulation, protein extracts were analyzed for expression of Endoglin (with an antibody that is specific for mouse Endoglin), TGF-β receptor II (RII), Fibronectin, ColI, vimentin, α-SMA, CTGF, Id2, and GFP by Western blot. Membranes (**A, C**) were incubated with an antibody to β-actin to monitor equal protein loading**.** Band intensities of the indicated proteins (**A, C**) were measured, normalized to β-actin and represented as fold induction of the unstimulated sample. The depicted results show representative images of one of three independent experiments.

In a next step we characterized the optimal time points for short-term responses (30 min or 1 h) and long-term responses (48 hrs) as a prerequisite for endoglin analysis (see below). Upon short-term stimulation, Smad1/5/8 as well as Smad2/Smad3 were activated at both TGF-β1 concentrations tested (Figure 5A). In line, the Smad3 target gene CTGF was strongly and the Smad1/5/8 target genes Id1 and Id2 slightly increased. The MAP kinases p38 and ERK1/2 were also activated at both concentrations of TGF-β1. In addition, the linker region of Smad2 (Smad2L) was phosphorylated in response to TGF-β1 application, reflecting the integration of ALK5 and MAP kinase signalling (data not shown). All of these responses are dependent on ALK5 (sensitivity to SB431542) but are not affected by the BMP-receptor inhibitor Dorsomorphin (DM). In addition, the analysis revealed that ALK5 is expressed in these cells (Figure S4). Since Dorsomorphin had no impact on TGF-β1 signalling (see Figure 5; Figure S5 and S6), the expression of other BMP-type ALKs including ALK1 is most likely not of functional relevance.

The densitometric analysis (Figure S7A) revealed that TGF-β induced phosphorylation of Smad1/5/8 (up to tenfold), its target gene Id2 (up to fivefold), and phosphorylation of Smad2 (fortyfold) and its target gene CTGF (up to five fold). All responses were markedly blocked by SB-431542 (SB). MAP kinase (i.e. p38 and p42) were induced to fourfold by TGF-β1.

BMP-7 on the other hand activates MAP kinases in a similar manner compared to TGF-β1, but primarily leads to phosphorylation of Smad1/5/8 and only a faint activation of Smad2/Smad3. The transcriptional activity of Smad3 and dependency on ALK5 in response to TGF-β1 is also shown by the (CAGA)_12_-MLP-Luc reporter (Figure 5B).

In response to 48 hrs stimulation with TGF-β1, the matrix proteins fibronectin and collagen I, the activation marker α-SMA and the pro-fibrogenic protein CTGF are concentration-dependently induced (Figure 5C, *left panel*). In addition, GFP expression resembled the pattern of ColI. Id2 expression is inversely regulated to the before mentioned proteins, being down-regulated by TGF-β1 (1 ng/ml) which can be partially blocked by the ALK5 inhibitor SB431542. To further increase the signal-to-noise ratio for TGF-β1-responses especially for GFP induction, we applied the ALK5 inhibitor in one set of experiments already during starvation phase (16 hrs before induction, Figure 5C, *right panel*). This treatment lowered the expression of α-SMA and increased the expression of Id2 (compare lanes 1 and 6). Surprisingly, the TGF-β1-effect was reduced. The expression of the TGF-β1-receptors endoglin and TRII was increased by application of SB431542. Unexpectedly Collagen I, α-SMA and Id2 are higher expressed when cells were cultured transiently (starvation) in medium containing 0.5% FCS (Figure 5C, lanes 1, 6) compared to cells cultured under stimulation conditions (0.2% FCS, Figure 5C, lanes 2, 8).

Densitometric analysis demonstrated that in this experiment the basal expression of all genes analysed (i.e. Col I, α-SMA) was markedly reduced upon serum starvation (up to 80%) and induced after addition of TGF-β1 up to eightfold (Figure S7B). The gene that showed highest stimulation after addition of TGF-β1 was CTGF (up to fourteen fold). Again, the stimulatory effect of TGF-β1 was inhibited by SB-431542.

### Endoglin Expression in CoI-GFP Cells

**Figure 6:**
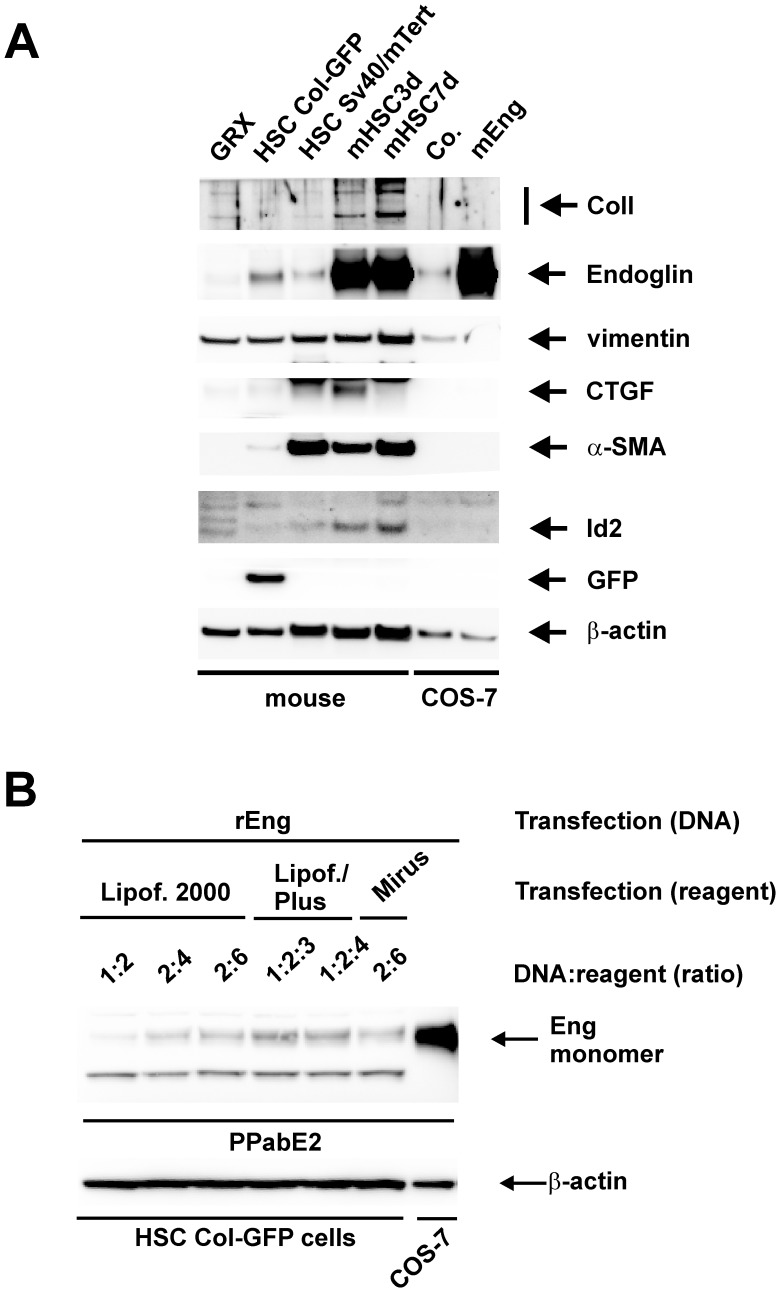
Endogenous and heterologous expression of endoglin. (**A**) The indicated mouse cells were cultured in growth medium (see Fig. 2F) and cellular proteins were analyzed by Western blot using specific antibodies to Collagen I, Endoglin (mouse specific), vimentin, CTGF, α-SMA, Id2, and GFP. To validate antibody specificity proteins of COS-7 cells transiently transfected with a mouse endoglin cDNA (mEng) or empty pcDNA vector as a control (Co.) were analyzed in parallel. The experiment was repeated three times. (**B**) Col-GFP or COS-7 cells were transiently transfected with a cDNA coding for rat endoglin (rEng) using the indicated transfection reagents and DNA to reagent ratios. Cellular proteins of the corresponding cells were prepared and analyzed by Western blot using a specific antibody to rat endoglin (PPabE2, [24]. Membranes (**A, B**) were incubated with an antibody to β-actin to monitor equal protein loading.

Figure 2F already implies that the expression of endoglin is generally very high in primary HSCs and nearly undetectable in murine or rat cell lines. This is underscored in Figure 6A in which we used a mouse endoglin specific antibody. Compared to the very strong expression of endoglin in early primary (mHSC3d) and late (mHSC7d) activated HSCs, the endogenous expression of endoglin in the immortalized cell lines is almost undetectable. This low endoglin expression is most likely reflecting the reduced endoglin abundance observed in fully transdifferentiated primary MFB-like cells compared to activated primary HSCs that was reported by us previously [24].

Due to the functional properties of Col-GFP cells and the low expression of endoglin we reasoned that these cells might be an ideal system to analyze the impact of endoglin on pro-fibrogenic responses mediated by TGF-β1. Since transient transfection especially of primary cells but also in cell lines is not always applicable we first established the transient overexpression of rat Endoglin in Col-GFP cells using different transfection reagents and conditions (Figure 6B). We used rat Endoglin in these studies to differentiate endogenous (mouse) endoglin from exogenous (rat) Endoglin with our specific rat Endoglin antibody and because rat Endoglin was functionally characterized before in fibrogenic signalling in CFSC-2G cells [24]. Although transfection with the Lipofectamine 2000 reagent at a 2∶4 ratio did not result in the highest protein expression, it was amongst all reagents tested the best choice with respect to the culture conditions and was therefore used for the following experiments.

### Impact of Endoglin Overexpression in Col-GFP Cells on TGF-β1 Signalling and Marker Protein Expression

**Figure 7:**
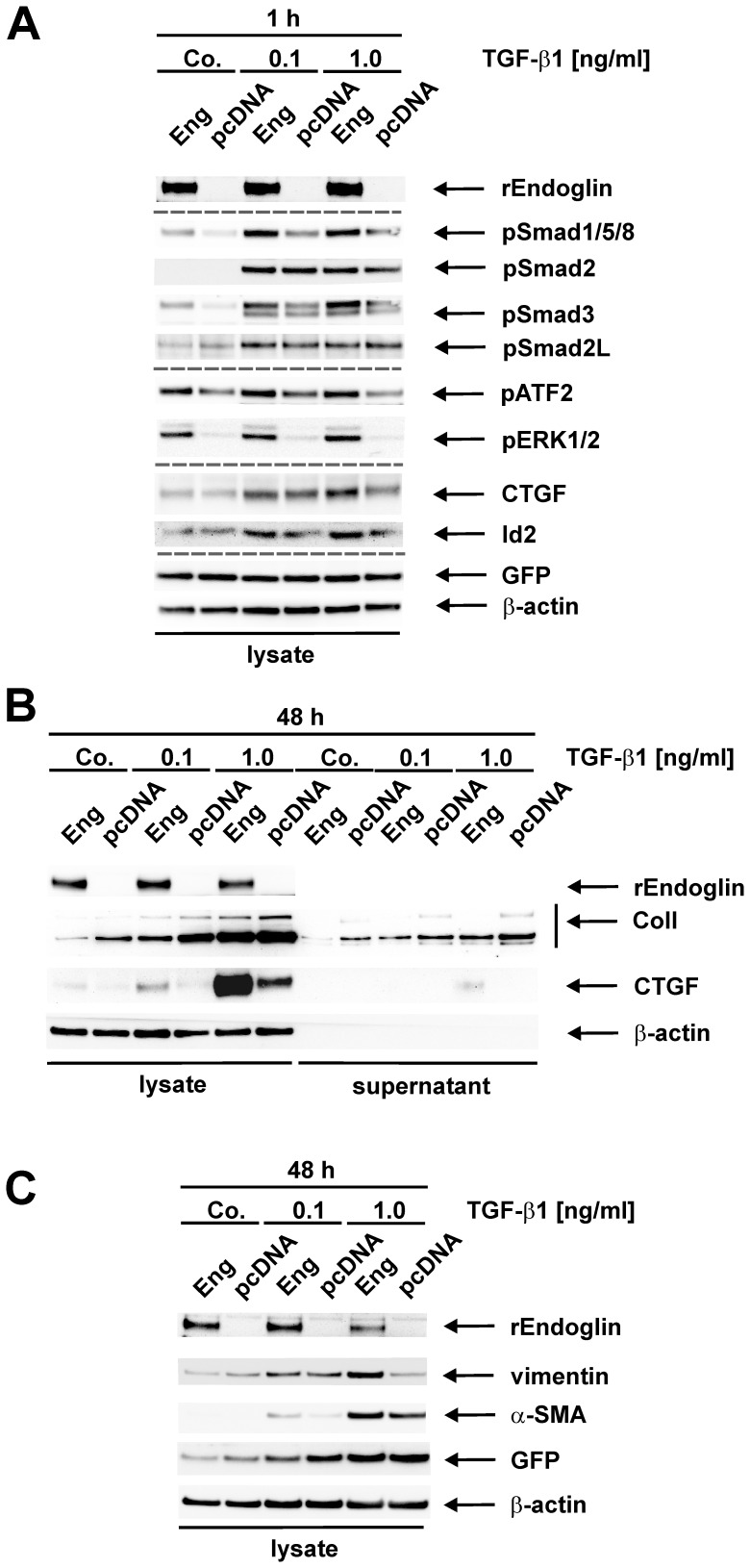
Effects of endoglin on TGF-β1 responses in Col-GFP cells. (**A**) Cells were transiently transfected with a cDNA for rat endoglin (Eng) or control vector (pcDNA). Thereafter, cells were stimulated with TGF-β1 (0.1 ng/ml; 1.0 ng/ml) or left untreated (Co.)). After the indicated time, cellular proteins were prepared and analyzed by Western blot using specific antibodies for rat endoglin (endoglin, PPabE2), phosphorylated Smad1/5/8 (pSmad1/5/8), C-terminally- (pSmad2) and linker-phosphorylated Smad2 (pSmad2L), phosphorylated Smad3 (pSmad3), the phosphorylated forms of ERK1/2 (pERK1/2), phosphorylated ATF-2 (pATF-2), CTGF, Id2, and GFP. (**B, C**) Cells were transiently transfected and stimulated with TGF-β1 or not as described in (**A**) for a time period of 48 hrs. Thereafter, cellular (**B, C**) and secreted (**B**) proteins were prepared and analyzed by Western blot using specific antibodies to endoglin, (**B**) Collagen I and CTGF, (**C**) vimentin, α-SMA and GFP. Membranes (**A–C**) were incubated with an antibody to β-actin to monitor equal protein loading. Results show representative images of one of three independent experiments.

To analyze the function of endoglin in TGF-β1 signalling in Col-GFP cells, we evaluated short-term (1 h) and long-term (48 hrs) responses (Figure 7A–C). With respect to Smad signalling, we only could demonstrate an enhancing effect on the Smad1/5/8 pathway, whereas the Smad2/Smad3 branch was not affected by endoglin overexpression (Figure 7A).

In addition to Smads, TGF-β1 induces the activation of the MAP kinases ERK1/2 and the substrate, e.g. activating transcription factor-2 (ATF-2). Both kinases are stronger phosphorylated in the presence of endoglin under basal or TGF-β1 inducing conditions. In line, stronger activation of Smad1/5/8 paralleled a slightly higher expression of the immediate early protein Id2. CTGF expression, which was shown to be dependent on ALK5 (Figure 5A, C) as well as regulated by ERK1/2 [24], [35], was also increased in the presence of endoglin. However, the phosphorylation of the linker region of Smad2 (pSmad2L) was only marginally (0.1 ng/ml TGF-β1) increased in the presence of endoglin (Figure 5A).

According to our published data [24], [35], we found that endoglin in long-term decreases expression and secretion of Collagen I as well as transgenic GFP transcriptionally controlled by the respective promoter (Figure 7B, C). In contrast, CTGF, vimentin and α-SMA were enhanced in the presence of endoglin (Figure 7B, C). Nevertheless, CTGF was only secreted at a very low level in the supernatant compared to collagen I (Figure 7B, *right panel*). In an additional experiment we could show that CTGF could be detected in the supernatant only in marginal amounts in the presence of TGF-β1 (1.0 ng/ml, 24 h). (Figure S8). This could be blocked by SB431542, similar to the results obtained in cell lysates (see Fig. 5C). This low secretion of CTGF was seen in all experiments performed (n = 3) and most likely reflects the low secretion rate of CTGF in HSC Col-GFP. Since all of these proteins depend on ALK5 activity (cf. Figure 5A, C), endoglin differentially modulates ALK5 based signal-transduction.

### Impact of Endoglin Overexpression in Col-GFP Cells on PDGF-BB-mediated ERK1/2 Activation

**Figure 8:**
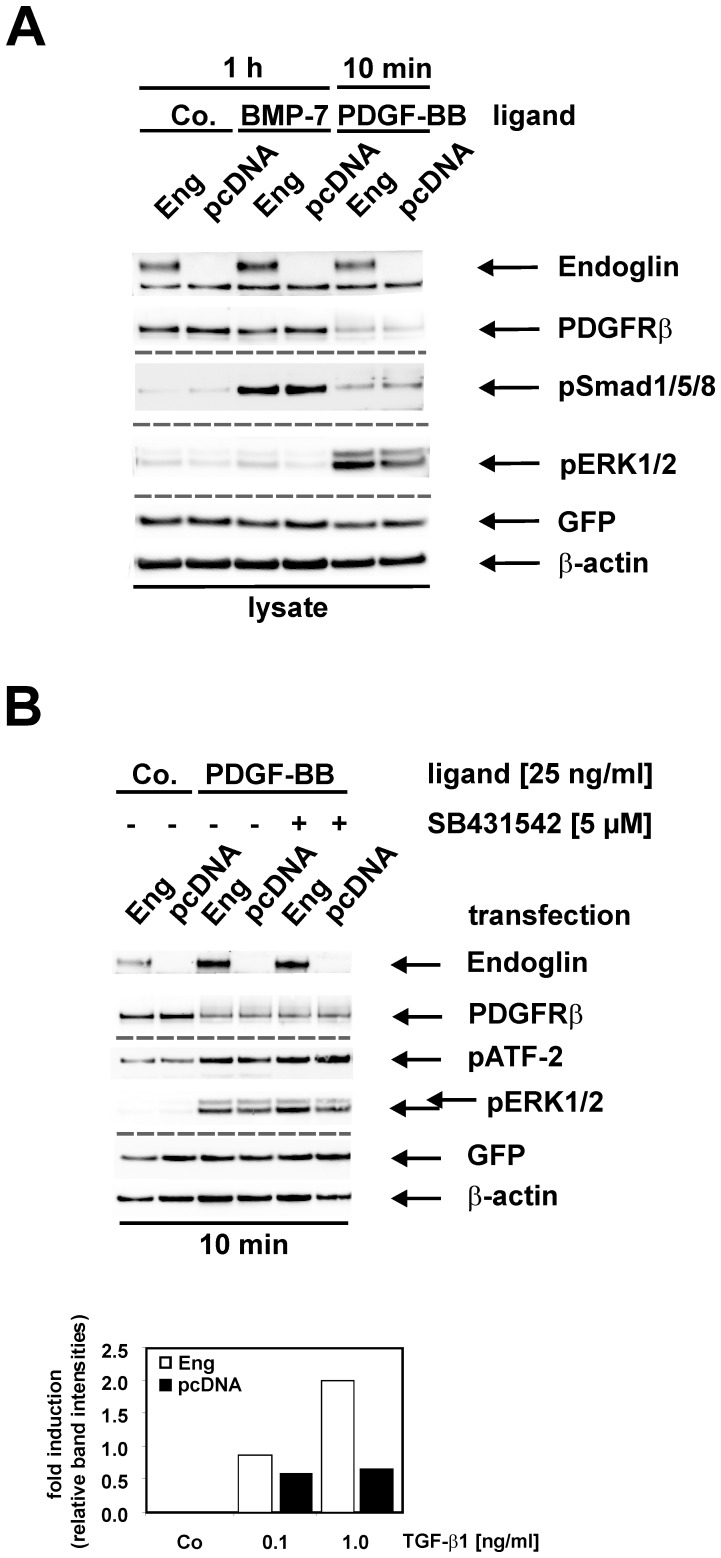
Endoglin effect on PDGF-BB signalling. (**A**) Cells were transiently transfected using Lipofectamine 2000 with a cDNA encoding rat endoglin. Thereafter, cells were stimulated with or without BMP-7 (25 ng/ml) for one hour or with PDGF-BB (25 ng/ml) for 10 min. Cellular proteins were prepared and analyzed in Western blot using specific antibodies to endoglin (PPabE2), PDGFRβ, phosphorylated Smad1/5/8 (pSmad1/5/8), phosphorylated ERK1/2 (pERK1/2) and GFP. (**B**) Cells were transiently transfected as described in (**A**) and stimulated or not with PDGF-BB (25 ng/ml) in the presence or absence of SB431542 (5 µM). Thereafter, cellular proteins were prepared and analyzed in Western blot using specific antibodies to endoglin (PPabE2), PDGFRβ, phosphorylated ATF-2 (pATF-2), phosphorylated ERK1/2 (pERK1/2) and GFP. All membranes were incubated with an antibody to β-actin to demonstrate equal protein loading. For densitometric analysis bands of β-actin and p-p42 were scanned and the latter one normalized to β-actin. Results are expressed as fold induction relative to the pcDNA control. Results show representative images of one of three independent experiments.

Endoglin was shown to enhance pSmad1/5/8 signalling in myofibroblast-like cells not only in response to TGF-β1, but also in response to BMP-7 [36]. In addition, since endoglin is able to modulate basal and TGF-β1-mediated ERK1/2 activation we asked if endoglin also could modulate ERK1/2 activation in response to a genuine ligand leading to ERK1/2 activation, i.e. PDGF-BB. As shown in Figure 8A BMP-7 causes a strong activation of Smad1/5/8 (see also Figure 5A) but endoglin has no effect on this activation (Figure 8A). In contrast, PDGF-BB mediated ERK1/2 activation is promoted in the presence of endoglin. Interestingly this effect is not dependent on ALK5 activity, because the ALK5-specific small inhibitor SB431542 does not interfere with the induction of ERK1/2 by PDGF-BB nor does it abolish the enhancing effect of endoglin (Figure 8B).

## Discussion

HSCs play an important role in the pathogenesis of liver fibrosis. Therefore, many features of HSC biology including expression of ECM, vitamin A storage, mechanisms of contractility and intracellular signalling are analyzed in primary HSC cultures. Primary HSCs undergo a complex cellular transition from quiescent cells to activated MFBs *in vitro*. On plastic dishes they transdifferentiate into MFBs within a few days. Several immortalized stellate cell lines have been also generated, but have many disadvantages. Because of the rapid transdifferentiation of quiescent HSCs into activated MFBs in culture during immortalization (usually about 3 weeks) HSC cell lines represent activated myofibroblasts rather than quiescent HSCs. As shown in Figure 2F and 2G, the different protein expression patterns in HSC cell lines are comparable to primary HSCs. Furthermore the Col-GFP cell line characterized here closely resembles the expression pattern observed for example in another immortalized established mouse HSC cell lines (i.e. GRX).

Moreover, the cellular origin of some cell lines is not entirely based on hepatic stellate cells. The widely used GRX cell line for example has been generated from fibrotic granulomas induced in C3H/HeN mouse liver by experimental infection with *Schistosoma mansoni* [37]. The widely used LX2 cell line [38] as well as the hTert cell line [5] have human origin and cannot be compared directly to murine stellate cells. Given the many different origins of MFBs in fibrotic liver (i.e. HSC, periportal fibroblasts, bone marrow derived cells [39], [40], [41], [42] it seems to be crucial to have a cell line with clearly defined cellular origin as close as possible to HSCs. Here we characterized an immortalized MFB cell line generated from quiescent HSC (Nycodenz centrifugation and microscopic analysis for Vitamin A droplets) derived from a mouse expressing GFP under the control of the collagen α1(I) promoter/enhancer [43]. In immunofluorescent-, mRNA- and Western blot-analysis we clearly show the expression of mesenchymal next to neurogenic markers as well as collagen expression, features distinctive for cells of HSC origin.

By using isolated quiescent HSC from this mouse, we not only immortalized HSCs derived MFBs but also used the endogenous property of these cells to express GFP upon activation. Therefore, *in vitro* these cells exhibit a strong GFP expression reflecting the activation of the collagen promoter/enhancer. This can be useful for direct detection of these cells by immunofluorescence or FACS analysis. Furthermore, the use of a fluorescence plate reader enables easy bulk analysis of cell proliferation in response to multiple stimuli. However, most likely due to the strong activation on plastic surfaces, GFP expression does not reflect the collagen expression directly. Compared to the endogenous collagen expression, which is strongly upregulated upon TGF-β1 treatment and downregulated in the presence of the ALK5 inhibitor SB421543, GFP expression is only marginally influenced by these treatments. Due to this stable expression, cells can be traced in co-cultures and animal experiments, since the experimental setting does not impact the marker expression.

In order to analyze cellular functions relevant for the fibrogenic process, we first evaluated some characteristics/hallmarks of TGF-β1 signalling in the isolated Col-GFP cells. Others and we have shown previously that TGF-β1 leads to activation of the Smad1/5/8 as well as Smad2/3 pathways in primary rat HSC and cell line CFSC-2G as well as in ongoing hepatic fibrogenesis [23], [24]. A similar activation pattern is also seen in the Col-GFP cell line: TGF-β1 induces the phosphorylation of both Smad pathways in a time and concentration dependent manner (cf. Figures 4 and 5). In line, the expression of the Smad3 target gene CTGF was strongly and the expression of the Smad1/5/8 target genes Id1 and Id2 slightly increased. Again this pattern followed Smad activation known from primary HSCs [44], [45]. In addition, the timed regulation of Id proteins (here shown for Id2), which are regulators of HSC function [45], could be displayed (Figures 4 and 5). At early time points (up to ∼1 h), TGF-β1 activates Id2 expression whereas from 2 hrs on (up to 48 hrs) Id2 expression is inhibited. Both effects are sensitive to SB421543, underscoring the role of ALK5 in these responses.

Beside the activation of Smad proteins, TGF-β1 provokes non-Smad signalling in Col-GFP cells. The MAP kinases p38 and ERK1/2 were activated in a time- and concentration-dependent manner. Both responses were ALK5 dependent as validated by usage of the specific inhibitor SB421543. In summary these experiments clearly demonstrate that the activation of signalling intermediates and target genes closely resembles the pattern found in primary cultures of HSC and reflect the close relation of Col-GFP cells to MFBs derived from quiescent HSCs.

In contrast to high expression of endoglin in primary activated HSC, the endogenous expression of endoglin in the immortalized cell lines is nearly undetectable. Usually completely transdifferentiated primary MFB-like cells do not express large quantities of endoglin [23], [24]. Because of the close stellate cell origin combined with the low expression of endoglin, we used these cells as a molecular tool to further investigate the impact of endoglin on pro-fibrogenic responses mediated by TGF-β1. By transient overexpressing rat endoglin in these cells, we were able to discriminate endogenous (mouse) endoglin from exogenous (rat) endoglin with specific antibodies. In line with the results obtained in CFSC-2G, the overexpression of endoglin in Col-GFP cells led to an enhanced activation of the Smad1/5/8 pathway, not affecting Smad2/3 signalling (short term) and increased expression of α-SMA (long term, cf. Figure 7A/B).

In addition to the Smad intracellular signalling mediators, TGF-β1 was shown to activate the MAP kinases ERK1/2 (see above). In the presence of endoglin, the phosphorylation of these kinases is clearly increased. Because this increase is also observed upon stimulation with PDGF-BB representing a strong activator of ERK1/2, the effect of endoglin on ERK1/2 seems to be a general phenomenon. An involvement of endoglin in regulating ERK1/2 activity has already consistently been shown in a few reports [46]–[49]. However, in contrast to the observation made in HSCs, endoglin inhibited ERK1/2 activation in other cell types except in T cells.

The positive effect of endoglin is even more interesting considering that in scleroderma fibroblasts the fibrogenic gene program is mediated *via* activation of Smad1 and ERK1/2 pathways [50], which are positively regulated by endoglin. Long-term stimulation with endoglin decreased cellular and secreted Collagen I levels as well as slightly transgenic GFP expression. In contrast, endoglin increased expression of CTGF and vimentin (in addition to α-SMA), responses that are ALK5 dependent. Endoglin therefore differentially modulates ALK5-based signal transduction and most likely changes pathways leading to HSC activation and transdifferentiation triggering fibrogenic responses in the liver.

Future work will show how useful the novel immortalized Col-GFP HSC cell line will be. Several potential *in vitro* applications will be the monitoring of therapeutic drugs in a reliable, easy to handle cell culture system that resembles primary HSC/MFB, the set up of co-culture systems in which the different cells incorporated should be easily discriminated, or the analysis of profibrogenic signalling cascades under defined conditions. In addition, the cell line will allow cell-tracing experiments in which different aspects of homing, lifetime, and reversibility of fibrosis or even more involvement of HSC/MFB in tumorgenesis could be addressed.

## Supporting Information

Figure S1 **Repetitions of experiment shown in** **Figure 4A** **.**
(TIF)

Figure S2 **Repetitions of experiment shown in** **Figure 4B** **.**
(TIF)

Figure S3 **Densitometric analysis of one representative experiment shown in** **Figure 4** **.**
(TIF)

Figure S4 **Expression of ALK5 in HSC Col-GFP. (A)** To demonstrate the expression of ALK5 in HSC Col-GFP cells were cultured in growth medium (GM), or were starved (0.5% FCS) and treated with 10% FCS, TGF-β1 (1.0 ng/ml) or left untreated (Co.) for the indicated times (30 min, 48 h). Thereafter, cellular proteins were extracted and analysed by Western blot using specific antibodies to the TGF-β-receptors Betaglycan (TGFβRIII, glycosylated form), TGFβRII and TGFβRI (ALK5). As a control for TGF-β1 application, phosphorylated Smad2 (short term, 30 min) or α-SMA (long term, 48 h) was analysed. **(B)** To further demonstrate ALK5 expression in the presence of various inhibitors, cells were starved and either not treated (Co.) or stimulated with TGF-β1 (1.0 ng/ml) in the presence of the indicated substances. Thereafter, cellular proteins were extracted and analysed by Western blot using specific antibodies to the TGF-β-receptors Betaglycan (TGFβRIII, glycosylated form), TGFβRII, and TGFβRI (ALK5). As a control for TGF-β1 activity and to monitor the effect of SB431542, the expression of α-SMA and CTGF expression was analysed. Both proteins are induced by TGF-β1 and this effect is abrogated in the presence of SB431542. Dorsomorphin (DM) was not effective since this substance does not influence Smad activation in this experimental setting (see also Fig. 5A). In conclusion, all three TGF-β receptors are expressed in HSC Col-GFP and ALK5 is present under all tested conditions.
(TIF)

Figure S5 **Repetitions of experiments shown in** **Figure 5A** **.**
(TIF)

Figure S6 **Repetitions of experiments shown in** **Figure 5C** **.**
(TIF)

Figure S7 **Densitometric analysis of experiments shown in** **Figure 5A and 5C** **.**
(TIF)

Figure S8 **Secretion of CTGF in HSC Col-GFP.** In an additional experiment that was done to demonstrate CTGF secretion, CTGF could be detected in the supernatant only in marginal amounts in the presence of TGF-β1 (1.0 ng/ml, 24 h). The abundance in the supernatant was blocked in the presence of SB431542, similar to the results obtained in cell lysates (see Fig. 5C). In comparison, ColI is secreted in higher amounts, increased by TGF-β1 (1.0 ng/ml, 24 h), and decreased in the presence of SB431542. In Figure 7B detection of the secreted CTGF protein in supernatants was very low and only visible when using 1.0 ng/ml TGF-β1 in the presence of Endoglin. This low secretion of CTGF was seen in all experiments performed (n = 3) and most likely reflects the low secretion rate of CTGF in HSC Col-GFP.
(TIF)

Table S1 **Cells and media used in this study.**
(DOC)

Table S2 **Antibodies used in this study.**
(DOC)

Table S3 **Primers and cycle conditions used in this study.**
(DOC)

## References

[1] Bataller R, Brenner DA (2005) Liver fibrosis. J Clin Invest 115: 209–218. 15690074 10.1172/JCI24282 PMC546435

[2] Friedman SL (2008) Hepatic stellate cells: protean, multifunctional, and enigmatic cells of the liver. Physiol Rev 88: 125–172. 18195085 10.1152/physrev.00013.2007 PMC2888531

[3] Herrmann J, Gressner AM, Weiskirchen R (2007) Immortal hepatic stellate cell lines: useful tools to study hepatic stellate cell biology and function? J Cell Mol Med 11: 704–722. 17760834 10.1111/j.1582-4934.2007.00060.x PMC3823251

[4] Xu L, Hui AY, Albanis E, Arthur MJ, O’Byrne SM, et al (2005) Human hepatic stellate cell lines, LX-1 and LX-2: new tools for analysis of hepatic fibrosis. Gut 54: 142–151. 15591520 10.1136/gut.2004.042127 PMC1774377

[5] Schnabl B, Choi YH, Olsen JC, Hagedorn CH, Brenner DA (2002) Immortal activated human hepatic stellate cells generated by ectopic telomerase expression. Lab Invest 82: 323–333. 11896211 10.1038/labinvest.3780426

[6] Blaner WS, O’Byrne SM, Wongsiriroj N, Kluwe J, D’Ambrosio DM, et al (2009) Hepatic stellate cell lipid droplets: a specialized lipid droplet for retinoid storage. Biochim Biophys Acta 1791: 467–473. 19071229 10.1016/j.bbalip.2008.11.001 PMC2719539

[7] Asahina K, Tsai SY, Li P, Ishii M, Maxson RE Jr, et al (2009) Mesenchymal origin of hepatic stellate cells, submesothelial cells, and perivascular mesenchymal cells during mouse liver development. Hepatology 49: 998–1011. 19085956 10.1002/hep.22721 PMC2673117

[8] Cao Y, Szabolcs A, Dutta SK, Yaqoob U, Jagavelu K, et al (2010) Neuropilin-1 mediates divergent R-Smad signaling and the myofibroblast phenotype. J Biol Chem 285: 31840–31848. 20675371 10.1074/jbc.M110.151696 PMC2951255

[9] Wynn T (2008) Cellular and molecular mechanisms of fibrosis. J Pathol 214: 199–210. 18161745 10.1002/path.2277 PMC2693329

[10] Brenner DA (2009) Molecular pathogenesis of liver fibrosis. Trans Am Clin Climatol Assoc 120: 361–368. 19768189 PMC2744540

[11] Blobe GC, Schiemann WP, Lodish HF (2000) Role of transforming growth factor beta in human disease. N Engl J Med 342: 1350–1358. 10793168 10.1056/NEJM200005043421807

[12] Kanzler S, Lohse AW, Keil A, Henninger J, Dienes HP, et al (1999) TGF-beta1 in liver fibrosis: an inducible transgenic mouse model to study liver fibrogenesis. Am J Physiol 276: G1059–1068. 10198351 10.1152/ajpgi.1999.276.4.G1059

[13] Sato M, Muragaki Y, Saika S, Roberts AB, Ooshima A (2003) Targeted disruption of TGF-beta1/Smad3 signaling protects against renal tubulointerstitial fibrosis induced by unilateral ureteral obstruction. J Clin Invest 112: 1486–1494. 14617750 10.1172/JCI19270 PMC259132

[14] Orlova VV, Liu Z, Goumans MJ, ten Dijke P (2011) Controlling angiogenesis by two unique TGF-beta type I receptor signaling pathways. Histol Histopathol 26: 1219–1230. 21751154 10.14670/HH-26.1219

[15] Uchinami H, Seki E, Brenner DA, D’Armiento J (2006) Loss of MMP 13 attenuates murine hepatic injury and fibrosis during cholestasis. Hepatology 44: 420–429. 16871591 10.1002/hep.21268

[16] Shi Y, Massague J (2003) Mechanisms of TGF-beta signaling from cell membrane to the nucleus. Cell 113: 685–700. 12809600 10.1016/s0092-8674(03)00432-x

[17] Gougos A, Letarte M (1990) Primary structure of endoglin, an RGD-containing glycoprotein of human endothelial cells. J Biol Chem 265: 8361–8364. 1692830

[18] Bernabeu C, Conley BA, Vary CP (2007) Novel biochemical pathways of endoglin in vascular cell physiology. J Cell Biochem 102: 1375–1388. 17975795 10.1002/jcb.21594 PMC2199238

[19] Guerrero-Esteo M, Lastres P, Letamendia A, Perez-Alvarez MJ, Langa C, et al (1999) Endoglin overexpression modulates cellular morphology, migration, and adhesion of mouse fibroblasts. Eur J Cell Biol 78: 614–623. 10535303 10.1016/S0171-9335(99)80046-6

[20] Meurer SK, Tihaa L, Lahme B, Gressner AM, Weiskirchen R (2005) Identification of endoglin in rat hepatic stellate cells: new insights into transforming growth factor beta receptor signaling. J Biol Chem 280: 3078–3087. 15537649 10.1074/jbc.M405411200

[21] Velasco S, Alvarez-Munoz P, Pericacho M, Dijke PT, Bernabeu C, et al (2008) L- and S-endoglin differentially modulate TGFbeta1 signaling mediated by ALK1 and ALK5 in L6E9 myoblasts. J Cell Sci 121: 913–919. 18303046 10.1242/jcs.023283

[22] Diez-Marques L, Ortega-Velazquez R, Langa C, Rodriguez-Barbero A, Lopez-Novoa JM, et al (2002) Expression of endoglin in human mesangial cells: modulation of extracellular matrix synthesis. Biochim Biophys Acta 1587: 36–44. 12009422 10.1016/s0925-4439(02)00051-0

[23] Clemente M, Nunez O, Lorente R, Rincon D, Matilla A, et al (2006) Increased intrahepatic and circulating levels of endoglin, a TGF-beta1 co-receptor, in patients with chronic hepatitis C virus infection: relationship to histological and serum markers of hepatic fibrosis. J Viral Hepat 13: 625–632. 16907850 10.1111/j.1365-2893.2006.00733.x

[24] Meurer SK, Tihaa L, Borkham-Kamphorst E, Weiskirchen R (2011) Expression and functional analysis of endoglin in isolated liver cells and its involvement in fibrogenic Smad signalling. Cell Signal 23: 683–699. 21146604 10.1016/j.cellsig.2010.12.002

[25] Yagmur E, Rizk M, Stanzel S, Hellerbrand C, Lammert F, et al (2007) Elevation of endoglin (CD105) concentrations in serum of patients with liver cirrhosis and carcinoma. Eur J Gastroenterol Hepatol 19: 755–761. 17700260 10.1097/MEG.0b013e3282202bea

[26] Schafer S, Zerbe O, Gressner AM (1987) The synthesis of proteoglycans in fat-storing cells of rat liver. Hepatology 7: 680–687. 3111965 10.1002/hep.1840070411

[27] Weiskirchen R, Gressner AM (2005) Isolation and culture of hepatic stellate cells. Methods Mol Med 117: 99–113. 16118448 10.1385/1-59259-940-0:099

[28] Tacke F, Weiskirchen R (2012) Update on hepatic stellate cells: pathogenic role in liver fibrosis and novel isolation techniques. Expert Rev Gastroenterol Hepatol 6: 67–80. 22149583 10.1586/egh.11.92

[29] Dennler S, Goumans MJ, ten Dijke P (2002) Transforming growth factor beta signal transduction. J Leukoc Biol 71: 731–740. 11994497

[30] Meurer SK, Lahme B, Tihaa L, Weiskirchen R, Gressner AM (2005) N-acetyl-L-cysteine suppresses TGF-beta signaling at distinct molecular steps: the biochemical and biological efficacy of a multifunctional, antifibrotic drug. Biochem Pharmacol 70: 1026–1034. 16098950 10.1016/j.bcp.2005.07.001

[31] Cassiman D, van Pelt J, De Vos R, Van Lommel F, Desmet V, et al (1999) Synaptophysin: A novel marker for human and rat hepatic stellate cells. Am J Pathol 155: 1831–1839. 10595912 10.1016/S0002-9440(10)65501-0 PMC1866940

[32] Wasmuth HE, Lammert F, Zaldivar MM, Weiskirchen R, Hellerbrand C, et al (2009) Antifibrotic Effects of CXCL9 and Its Receptor CXCR3 in Livers of Mice and Humans. Gastroenterology 137: 309–319.e303. 19344719 10.1053/j.gastro.2009.03.053 PMC2892869

[33] Kamada Y, Mori K, Matsumoto H, Kiso S, Yoshida Y, et al.. (2012) N-Acetylglucosaminyltransferase V regulates TGF-beta response in hepatic stellate cells and the progression of steatohepatitis. Glycobiology. 10.1093/glycob/cws012 22294551

[34] Dooley S, Delvoux B, Lahme B, Mangasser-Stephan K, Gressner AM (2000) Modulation of transforming growth factor beta response and signaling during transdifferentiation of rat hepatic stellate cells to myofibroblasts. Hepatology 31: 1094–1106. 10796885 10.1053/he.2000.6126

[35] Meurer SK, Esser M, Tihaa L, Weiskirchen R (2012) BMP-7/TGF-beta1 signalling in myoblasts: Components involved in signalling and BMP-7-dependent blockage of TGF-beta-mediated CTGF expression. Eur J Cell Biol 91: 450–463. 22099397 10.1016/j.ejcb.2011.09.004

[36] Scherner O, Meurer SK, Tihaa L, Gressner AM, Weiskirchen R (2007) Endoglin differentially modulates antagonistic transforming growth factor-beta1 and BMP-7 signaling. J Biol Chem 282: 13934–13943. 17376778 10.1074/jbc.M611062200

[37] Borojevic R, Monteiro AN, Vinhas SA, Domont GB, Mourao PA, et al (1985) Establishment of a continuous cell line from fibrotic schistosomal granulomas in mice livers. In Vitro Cell Dev Biol 21: 382–390. 4030623 10.1007/BF02623469

[38] Lee JS, Kang Decker N, Chatterjee S, Yao J, Friedman S, et al (2005) Mechanisms of nitric oxide interplay with Rho GTPase family members in modulation of actin membrane dynamics in pericytes and fibroblasts. Am J Pathol 166: 1861–1870. 15920170 10.1016/S0002-9440(10)62495-9 PMC1602419

[39] Iwaisako K, Brenner DA, Kisseleva T (2012) What’s new in liver fibrosis? The origin of myofibroblasts in liver fibrosis. J Gastroenterol Hepatol 27 Suppl 2 65–68. 22320919 10.1111/j.1440-1746.2011.07002.x PMC4841268

[40] Kisseleva T, Brenner DA (2007) Role of hepatic stellate cells in fibrogenesis and the reversal of fibrosis. J Gastroenterol Hepatol 22 Suppl 1 S73–78. 17567473 10.1111/j.1440-1746.2006.04658.x

[41] Dudas J, Mansuroglu T, Batusic D, Saile B, Ramadori G (2007) Thy-1 is an in vivo and in vitro marker of liver myofibroblasts. Cell Tissue Res 329: 503–514. 17576600 10.1007/s00441-007-0437-z

[42] Scholten D, Reichart D, Paik YH, Lindert J, Bhattacharya J, et al (2011) Migration of fibrocytes in fibrogenic liver injury. Am J Pathol 179: 189–198. 21703401 10.1016/j.ajpath.2011.03.049 PMC3123781

[43] Magness ST, Bataller R, Yang L, Brenner DA (2004) A dual reporter gene transgenic mouse demonstrates heterogeneity in hepatic fibrogenic cell populations. Hepatology 40: 1151–1159. 15389867 10.1002/hep.20427

[44] Jerkic M, Rivas-Elena JV, Santibanez JF, Prieto M, Rodriguez-Barbero A, et al (2006) Endoglin regulates cyclooxygenase-2 expression and activity. Circ Res 99: 248–256. 16840721 10.1161/01.RES.0000236755.98627.69

[45] Wiercinska E, Wickert L, Denecke B, Said HM, Hamzavi J, et al (2006) Id1 is a critical mediator in TGF-beta-induced transdifferentiation of rat hepatic stellate cells. Hepatology 43: 1032–1041. 16628634 10.1002/hep.21135

[46] Schmidt-Weber CB, Letarte M, Kunzmann S, Ruckert B, Bernabeu C, et al (2005) TGF-{beta} signaling of human T cells is modulated by the ancillary TGF-{beta} receptor endoglin. Int Immunol 17: 921–930. 15967783 10.1093/intimm/dxh272

[47] Rodriguez-Barbero A, Obreo J, Alvarez-Munoz P, Pandiella A, Bernabeu C, et al (2006) Endoglin modulation of TGF-beta1-induced collagen synthesis is dependent on ERK1/2 MAPK activation. Cell Physiol Biochem 18: 135–142. 16914898 10.1159/000095181

[48] Lee NY, Blobe GC (2007) The interaction of endoglin with beta-arrestin2 regulates transforming growth factor-beta-mediated ERK activation and migration in endothelial cells. J Biol Chem 282: 21507–21517. 17540773 10.1074/jbc.M700176200

[49] Santibanez JF, Perez-Gomez E, Fernandez LA, Garrido-Martin EM, Carnero A, et al (2010) The TGF-beta co-receptor endoglin modulates the expression and transforming potential of H-Ras. Carcinogenesis 31: 2145–2154. 20884686 10.1093/carcin/bgq199

[50] Pannu J, Nakerakanti S, Smith E, ten Dijke P, Trojanowska M (2007) Transforming growth factor-beta receptor type I-dependent fibrogenic gene program is mediated via activation of Smad1 and ERK1/2 pathways. J Biol Chem 282: 10405–10413. 17317656 10.1074/jbc.M611742200

