# Supplementary figures and images for: Overexpression of Endoglin Modulates TGF-β1-Signalling Pathways in a Novel Immortalized Mouse Hepatic Stellate Cell Line
Source: PLoS One. 2013 Feb 20;8(2):e56116. doi: 10.1371/journal.pone.0056116 (PMC3577806; doi:10.1371/journal.pone.0056116)

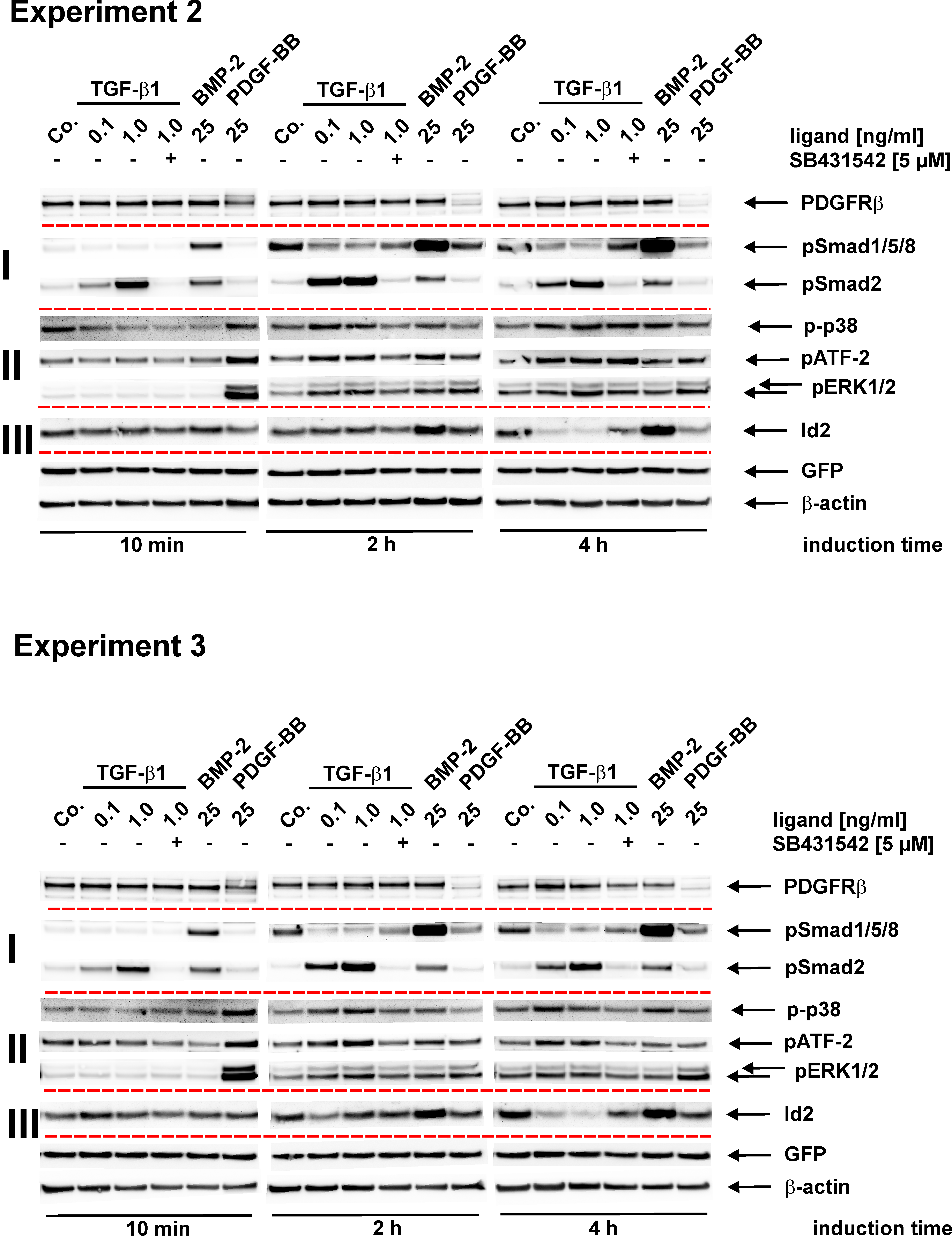

Supplement: Figure S1 — Repetitions of experiment shown in Figure 4A . (TIF) [file pone.0056116.s001.tif]

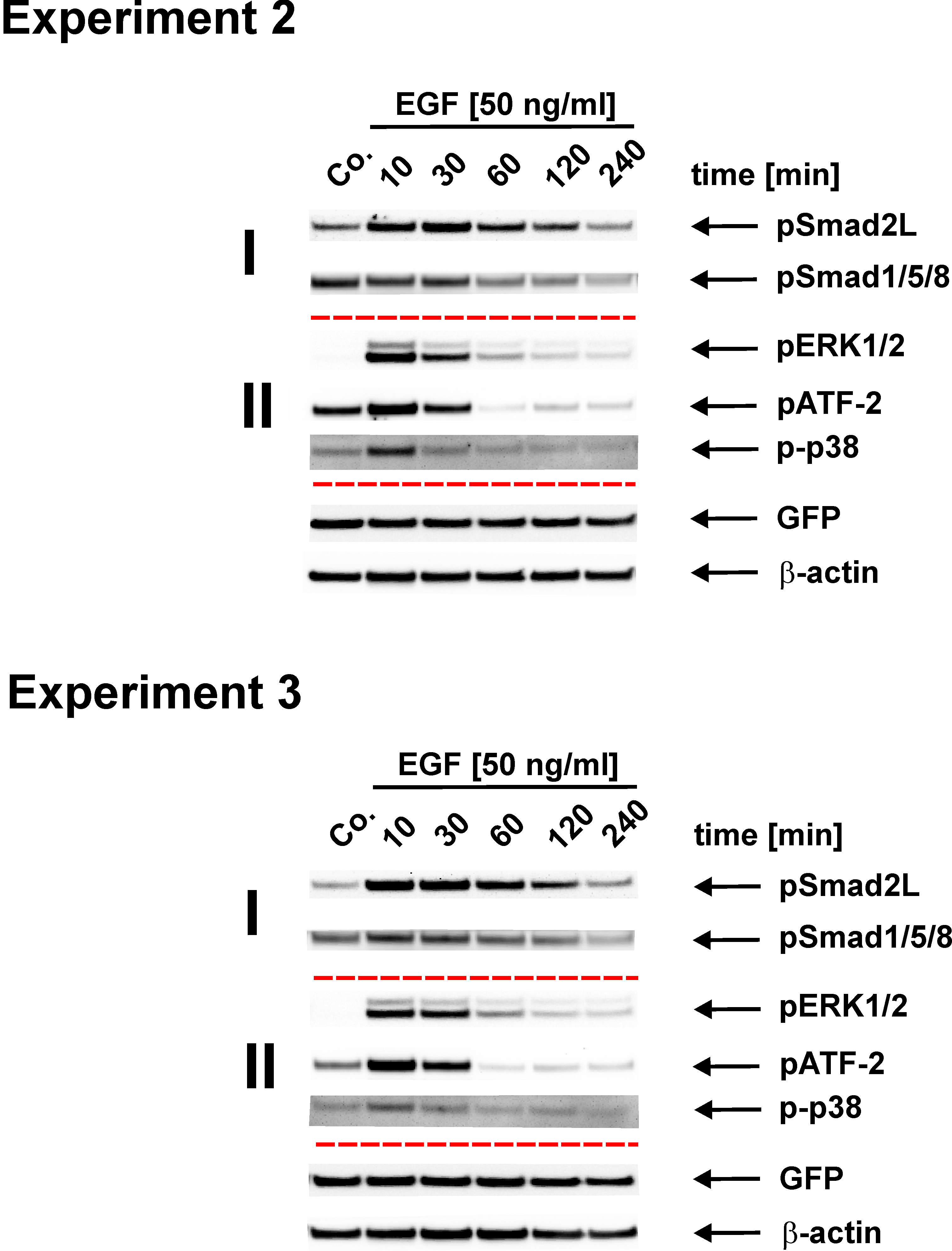

Supplement: Figure S2 — Repetitions of experiment shown in Figure 4B . (TIF) [file pone.0056116.s002.tif]

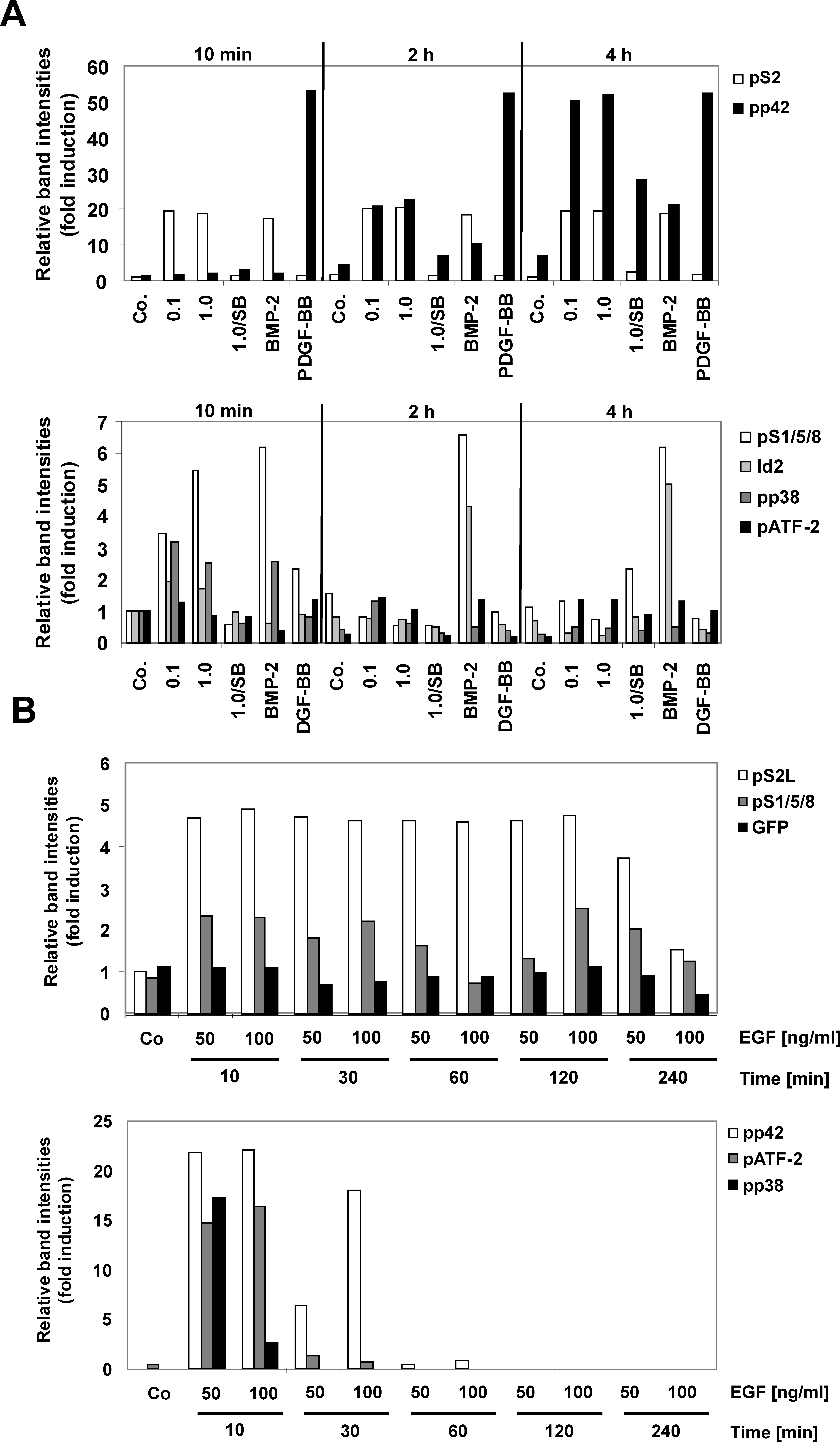

Supplement: Figure S3 — Densitometric analysis of one representative experiment shown in Figure 4 . (TIF) [file pone.0056116.s003.tif]

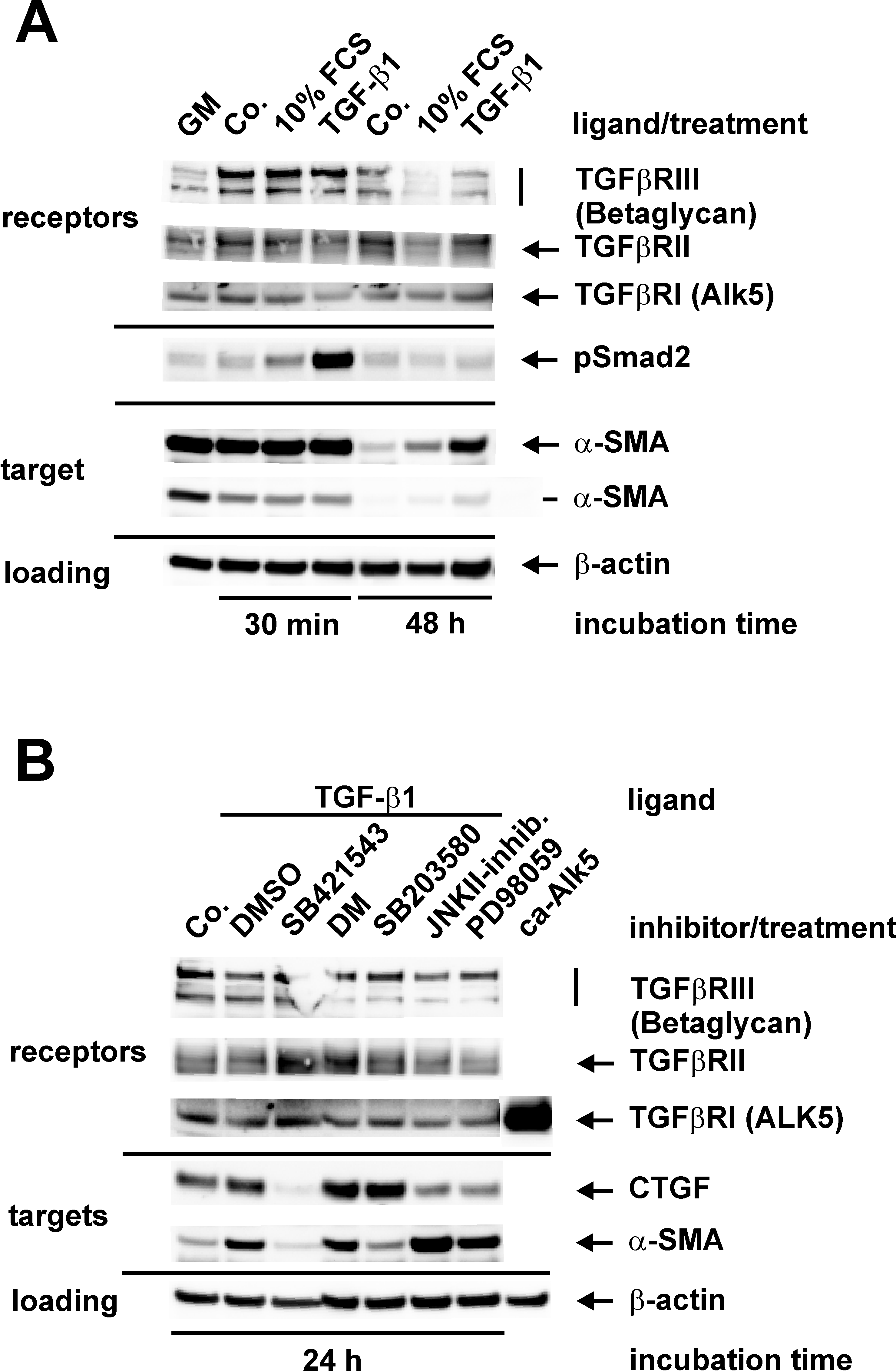

Supplement: Figure S4 — Expression of ALK5 in HSC Col-GFP. (A) To demonstrate the expression of ALK5 in HSC Col-GFP cells were cultured in growth medium (GM), or were starved (0.5% FCS) and treated with 10% FCS, TGF-β1 (1.0 ng/ml) or left untreated (Co.) for the indicated times (30 min, 48 h). Thereafter, cellular proteins were extracted and analysed by Western blot using specific antibodies to the TGF-β-receptors Betaglycan (TGFβRIII, glycosylated form), TGFβRII and TGFβRI (ALK5). As a control for TGF-β1 application, phosphorylated Smad2 (short term, 30 min) or α-SMA (long term, 48 h) was analysed. (B) To further demonstrate ALK5 expression in the presence of various inhibitors, cells were starved and either not treated (Co.) or stimulated with TGF-β1 (1.0 ng/ml) in the presence of the indicated substances. Thereafter, cellular proteins were extracted and analysed by Western blot using specific antibodies to the TGF-β-receptors Betaglycan (TGFβRIII, glycosylated form), TGFβRII, and TGFβRI (ALK5). As a control for TGF-β1 activity and to monitor the effect of SB431542, the expression of α-SMA and CTGF expression was analysed. Both proteins are induced by TGF-β1 and this effect is abrogated in the presence of SB431542. Dorsomorphin (DM) was not effective since this substance does not influence Smad activation in this experimental setting (see also Fig. 5A). In conclusion, all three TGF-β receptors are expressed in HSC Col-GFP and ALK5 is present under all tested conditions. (TIF) [file pone.0056116.s004.tif]

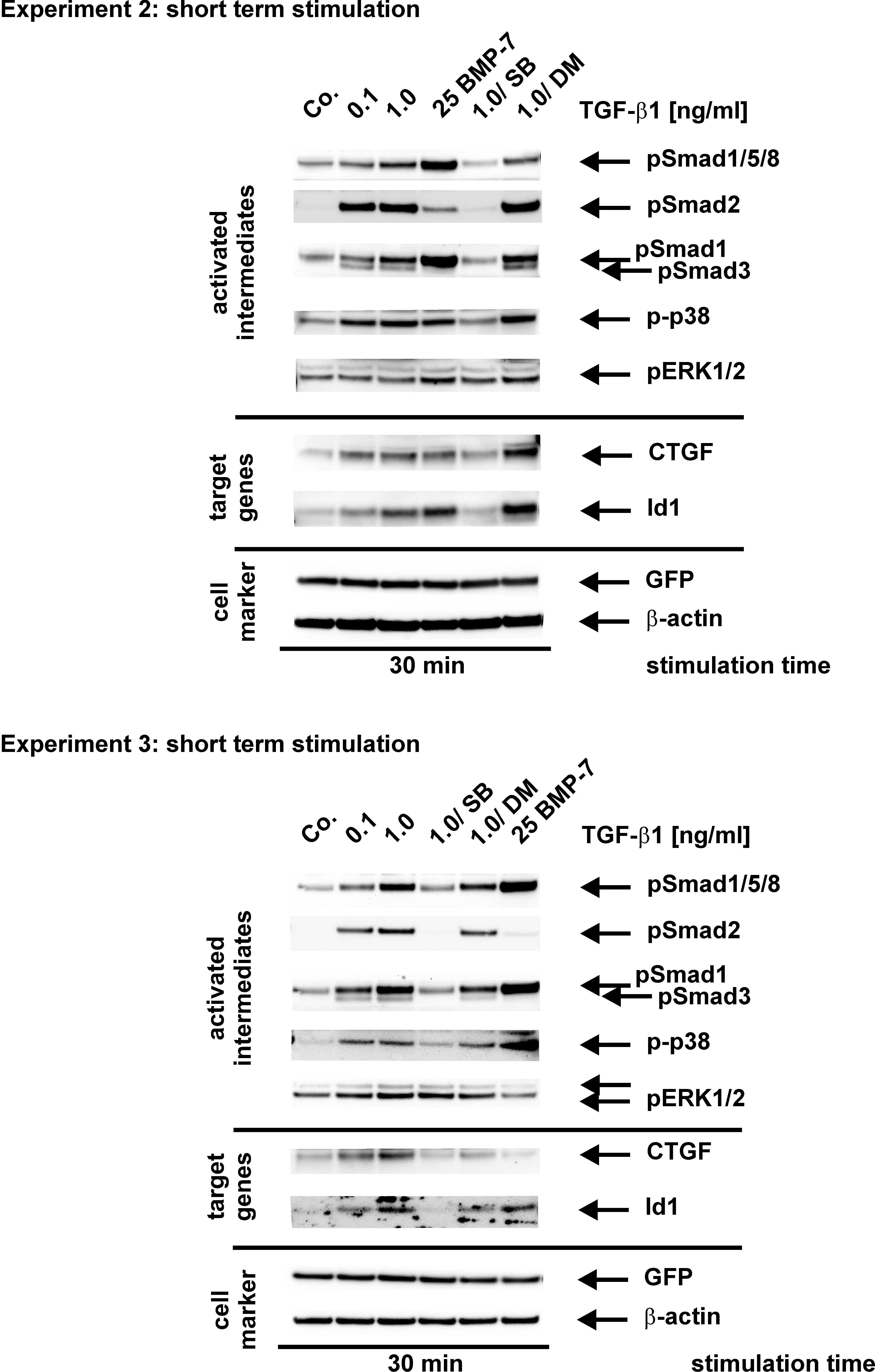

Supplement: Figure S5 — Repetitions of experiments shown in Figure 5A . (TIF) [file pone.0056116.s005.tif]

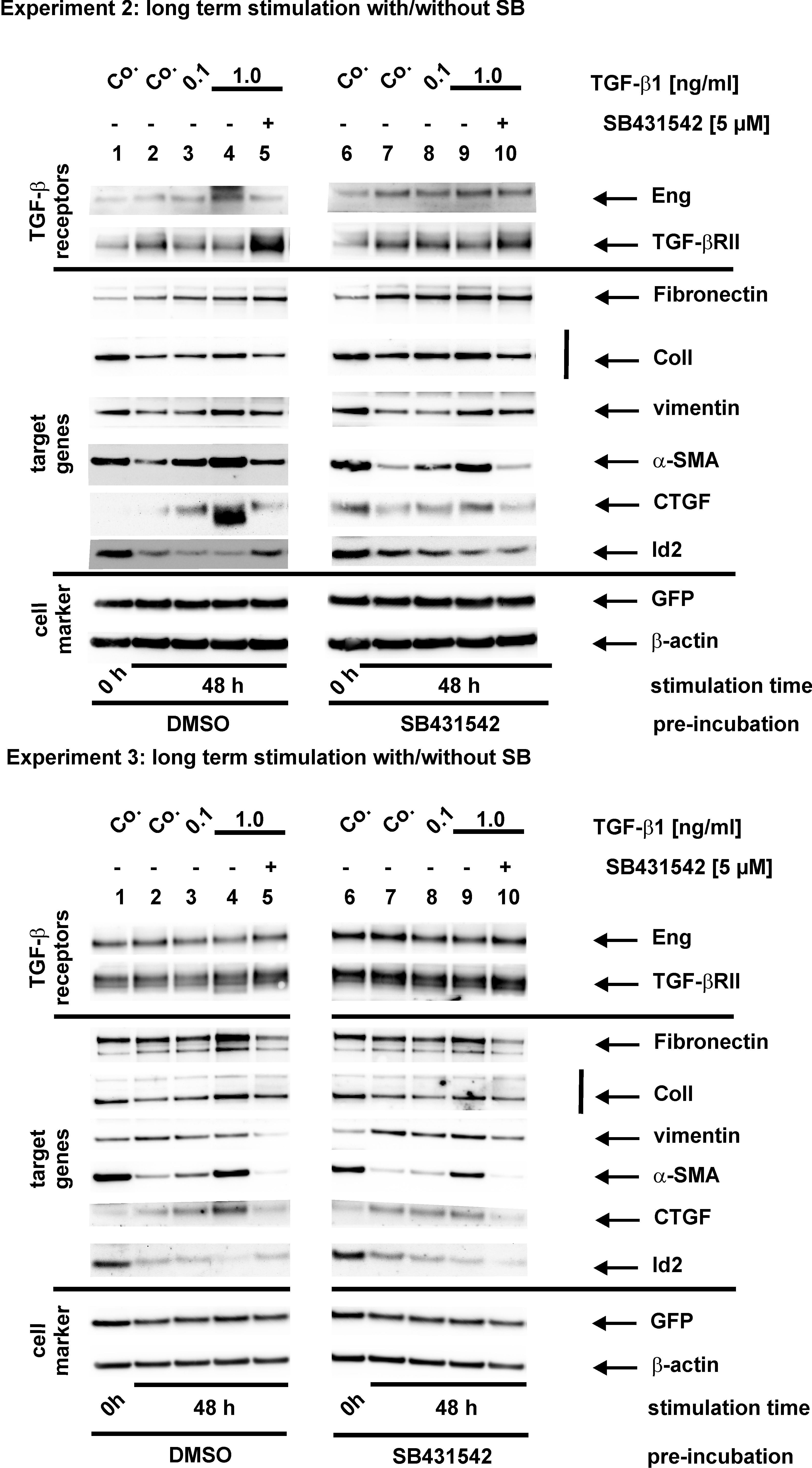

Supplement: Figure S6 — Repetitions of experiments shown in Figure 5C . (TIF) [file pone.0056116.s006.tif]

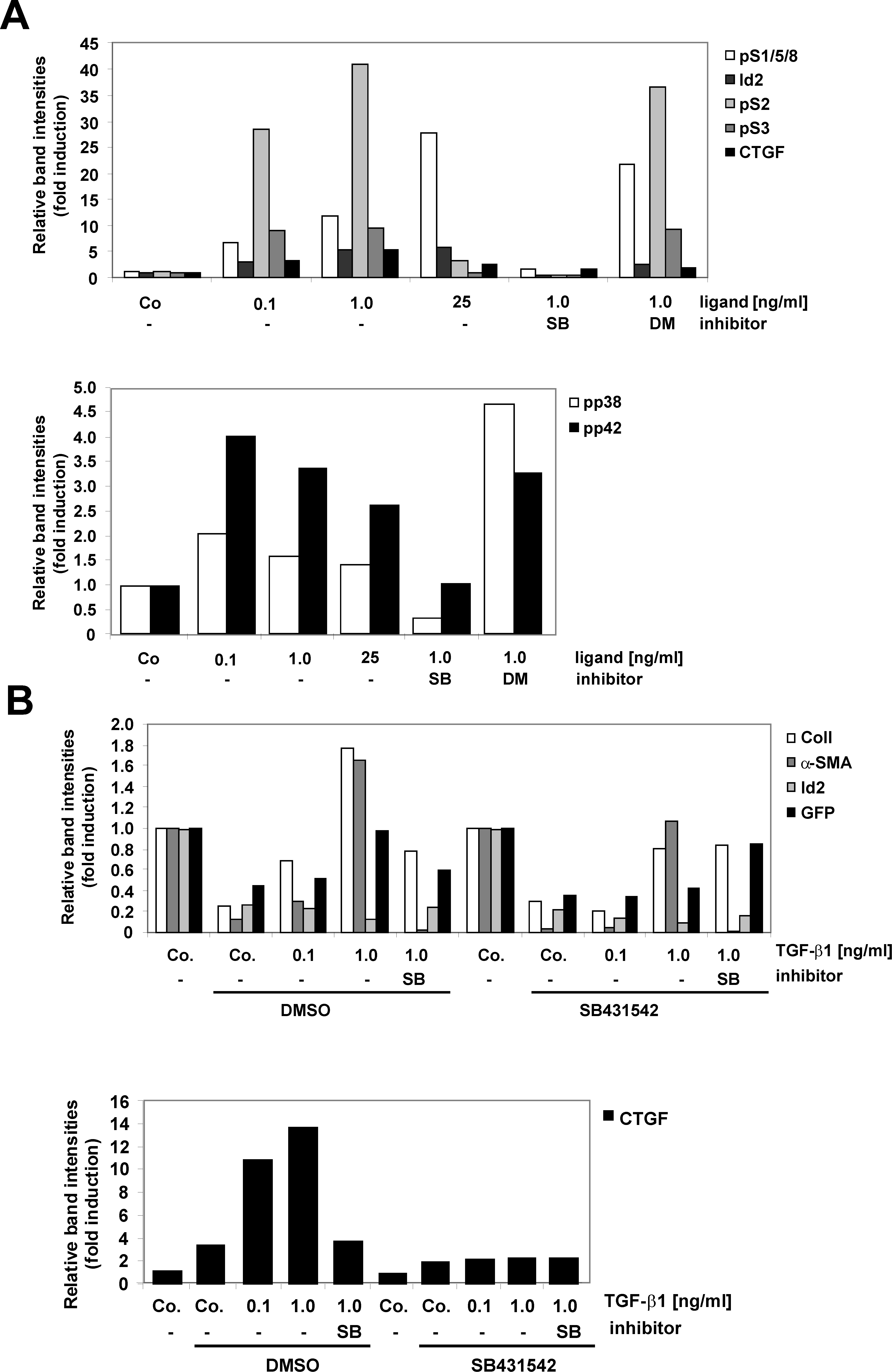

Supplement: Figure S7 — Densitometric analysis of experiments shown in Figure 5A and 5C . (TIF) [file pone.0056116.s007.tif]

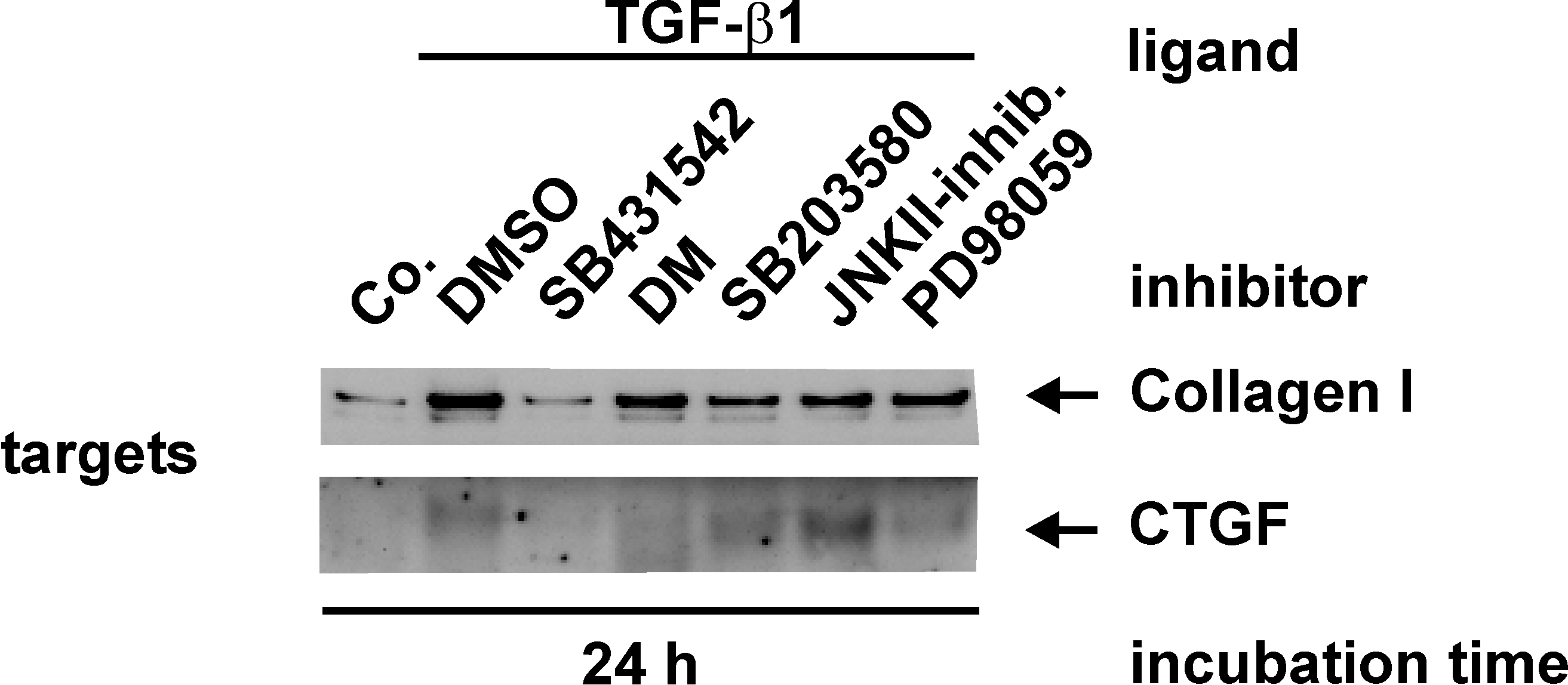

Supplement: Figure S8 — Secretion of CTGF in HSC Col-GFP. In an additional experiment that was done to demonstrate CTGF secretion, CTGF could be detected in the supernatant only in marginal amounts in the presence of TGF-β1 (1.0 ng/ml, 24 h). The abundance in the supernatant was blocked in the presence of SB431542, similar to the results obtained in cell lysates (see Fig. 5C). In comparison, ColI is secreted in higher amounts, increased by TGF-β1 (1.0 ng/ml, 24 h), and decreased in the presence of SB431542. In Figure 7B detection of the secreted CTGF protein in supernatants was very low and only visible when using 1.0 ng/ml TGF-β1 in the presence of Endoglin. This low secretion of CTGF was seen in all experiments performed (n = 3) and most likely reflects the low secretion rate of CTGF in HSC Col-GFP. (TIF) [file pone.0056116.s008.tif]
