# Supplementary material for: Overexpression of Endoglin Modulates TGF-β1-Signalling Pathways in a Novel Immortalized Mouse Hepatic Stellate Cell Line
Source: PLoS One. 2013 Feb 20;8(2):e56116. doi: 10.1371/journal.pone.0056116 (PMC3577806; doi:10.1371/journal.pone.0056116)
Supplement: Table S1 — Cells and media used in this study. (DOC) [file pone.0056116.s009.doc]

# Supplementary Table S1: Cells and media used in this study

| **Cell type** | **Origin** | **Ref** | **Medium*** | **Additives** |
| --- | --- | --- | --- | --- |
|  |  |  |  |  |
| **Primary cells** |  |  |  |  |
| HSC/MFB | rat | [1-3] | GM |  |
| HSC | mouse | [4] | GM |  |
| **Cell lines** |  |  |  |  |
| GRX | mouse | [5] | GM |  |
| HSC Col I-GFP | mouse | this work | GM |  |
| HSC SV40/mTert | mouse | this work | GM |  |
| CFSC-2G | rat | [6] | GM | 1% NEA** |
| HepG2 | human | [7] | RPMI |  |
| COS-7 | monkey | **ATCC** (CRL1651) | GM |  |

Note: * Growth medium (GM) is Dulbecco’s modified Eagle medium (DMEM) containing 10% FCS, 4 mM L-Glutamine, and 1 x antibiotics; ** NEA stands for non essential amino acids. RPMI contains 10% FCS, 4 mM L-Glutamine, and 1 x antibiotics.

References are:

1. Schafer S, Zerbe O, Gressner AM (1987) The synthesis of proteoglycans in fat-storing cells of rat liver. Hepatology 7: 680-687.
2. Fehrenbach H, Weiskirchen R, Kasper M, Gressner AM. (2001) Up-regulated expression of the receptor for advanced glycation end products in cultured rat hepatic stellate cells during transdifferentiation to myofibroblasts. Hepatology 34:943-952.
3. Weiskirchen R, Gressner AM (2005) Isolation and culture of hepatic stellate cells. Methods Mol Med 117: 99-113.
4. Tacke F, Weiskirchen R (2012) Update on hepatic stellate cells: pathogenic role in liver fibrosis and novel isolation techniques. Expert Rev Gastroenterol Hepatol 6: 67-80.
5. Borojevic R, Monteiro AN, Vinhas SA, Domont GB, Mourao PA, et al. (1985) Establishment of a continuous cell line from fibrotic schistosomal granulomas in mice livers. In Vitro Cell Dev Biol 21: 382-390.
6. Greenwel P, Schwartz M, Rosas M, Peyrol S, Grimaud JA, Rojkind M. (1991) Characterization of fat-storing cell lines derived from normal and CCl_4_-cirrhotic livers. Differences in the production of interleukin-6. Lab Invest. 65:644-653.
7. [Knowles BB](http://www.ncbi.nlm.nih.gov/pubmed?term=Knowles BB%5BAuthor%5D&cauthor=true&cauthor_uid=6248960), [Howe CC](http://www.ncbi.nlm.nih.gov/pubmed?term=Howe CC%5BAuthor%5D&cauthor=true&cauthor_uid=6248960), [Aden DP](http://www.ncbi.nlm.nih.gov/pubmed?term=Aden DP%5BAuthor%5D&cauthor=true&cauthor_uid=6248960). (1980) Human hepatocellular carcinoma cell lines secrete the major plasma proteins and hepatitis B surface antigen. [Science](http://www.ncbi.nlm.nih.gov/pubmed/?term=Human+hepatocellular carcinoma cell lines secrete the major plasma proteins and hepatitis B surface antigen." \l "%23) 209:497-499.
