# Supplementary material for: Overexpression of Endoglin Modulates TGF-β1-Signalling Pathways in a Novel Immortalized Mouse Hepatic Stellate Cell Line
Source: PLoS One. 2013 Feb 20;8(2):e56116. doi: 10.1371/journal.pone.0056116 (PMC3577806; doi:10.1371/journal.pone.0056116)
Supplement: Table S2 — Antibodies used in this study. (DOC) [file pone.0056116.s010.doc]

**Supplementary Table S2:**

**Antibodies used in this study**

|  |  |  |  |  |  |
| --- | --- | --- | --- | --- | --- |
| **Antibody** | **clonality** | **Supplied by** | **Epitope, location** | **Species** | **Dilution** |
| **TGF-**β **/ Endoglin / PDGF** | |  |  |  |  |
| sc-398 | Poly | Santa Cruz Biotech., Santa Cruz, CA, USA | Peptide mapping within a cytoplasmic domain of human TGFβ RI | h, m, r | 1:500 |
| sc-400 | Poly | Santa Cruz Biotech., Santa Cruz, CA, USA | Peptide mapping within a cytoplasmic domain of human TGFβ RII | h, m, r | 1:1,000 |
| AF242PB | Poly | R&D Systems, Wiesbaden, Germany | Recombinant human TGFβ RIII  Gly21-Asp781 | h, m | 1:1,000 |
| sc-6199 | Poly | Santa Cruz | Peptide mapping at the C-terminus of human TGFβ RIII | h, m, r | 1:500 |
| PPabE2 | Poly | Meurer et al., 2011 | Mapping at the N-terminus of rat Endoglin | r | 1:2,000 |
| AF1320 | Poly | R&D Systems, Wiesbaden, Germany | mouse Endoglin/CD105  Glu27Gly581 | m | 1:1,000 |
| sc-432 | Poly | Santa Cruz | Epitope corresponding to aa 958-1106 of human PDGF receptor type β | h, m, r | 1:1,000 |
| **MAP-kinases** | |  |  |  |  |
| 612281 | Mono | BD Biosciences, Heidelberg, Germany | Human p-p38 MAPK (pT180/pY182) | h, m, r | 1:1,000 |
| CS-9101 | Poly | Cell Signaling Technology, Danvers, MA, USA | Synthetic phospho-peptide corresponding to residues around Thr202/Tyr204of human p44 | h, m, r | 1:1,000 |
| **Smads** | |  |  |  |  |
| CS-9514* | Poly | Cell Signaling Technology | pSmad1/3, synthetic phospho-peptide hSmad3, Ser423/425 | h, m, r | 1:500 |
| CS-9511 | Poly | Cell Signaling Technology | pSmad1/5/8, synthetic phospho-peptide hSmad5, Ser463/465 | h, m, r | 1:1,000 |
| CS-3104 | Poly | Cell Signaling Technology | Synthetic phospho peptide corresponding to residues surrounding serines 245/250/255 of Smad2 | h, m, r | 1:1,000 |
| CS-3101 | Poly | Cell Signaling Technology | pSmad2, synthetic phospho-peptide hSmad2, Ser465/467 | h, m, r | 1:2,000 |
| **target proteins** | |  |  |  |  |
| sc-14939 | Poly | Santa Cruz | CTGF, internal region of hCTGF | h, m, r | 1:1,000 |
| ab-92547 | Mono | abcam, Cambridge, UK | A synthetic peptide corresponding to the C-terminus of human Vimentin | h, m, r | 1:1,000 |
| sc-488 | Poly | Santa Cruz | Id1, epitope mapping at the C-terminus, mouse origin | h, m, r | 1:500 |
| sc-489 | Poly | Santa Cruz | Id2, epitope mapping at the C-terminus, mouse origin | h, m, r | 1:500 |
| PS065 | Poly | Monosan, Hycultec GmbH, Beutelsbach, Germany | Rat Collagen I | h, m, r | 1:500 |
| AB1954 | Poly | Millipore, Schalbach, Germany | Purified rat plasma fibronectin | m, r | 1:1,000 |
| sc-7559 | Poly | Santa Cruz | Epitope mapping near the C-terminus of human Desmin | h, m, r | 1:500 |
| CS-9221 | Poly | Cell Signaling Technology | A synthetic phospho-peptide corresponding to residues surrounding Thr71 of human ATF-2 | h, m, r | 1:1,000 |
| sc-8334 | Poly | Santa Cruz | Full length GFP of *Aequorea victoria* | Aequorea victoria | 1:1,000 |
| sc-20800 | Poly | Santa Cruz | aa 4-30 of the SV40 Large T antigen | SV40 Large T antigen | 1:1,000 |
| [ab32127](http://www.abcam.com/Synaptophysin-antibody-YE269-ab32127.html) | Mono | abcam, Cambridge, UK | synthetic peptide corresponding to residues in C terminus (cytoplasmic domain) of human Synaptophysin | m, r, h | 1:1,000 |
| CBL-171 | Mono | Millipore | N-terminal amino acids of the α-smooth muscle isoform of actin | h, m, r | 1:1,000 |
| **protein loading** | |  |  |  |  |
| #A5441 | Mono | Sigma-Aldrich, Taufkirchen, Germany | β-actin, synthetic peptide N-terminus (clone AC-15) | h, m, r | 1:10,000 |

* Note: The antibody CS-9514 is not more available, it has been replaced by a monoclonal rabbit antibody (i.e. CS-9520) that was raised by immunizing rabbits with a synthetic KLH-coupled phospho-peptide corresponding to residues surrounding Ser423/425 of Smad3. This antibody does not cross-react with other family members.
