# Supplementary material for: Overexpression of Endoglin Modulates TGF-β1-Signalling Pathways in a Novel Immortalized Mouse Hepatic Stellate Cell Line
Source: PLoS One. 2013 Feb 20;8(2):e56116. doi: 10.1371/journal.pone.0056116 (PMC3577806; doi:10.1371/journal.pone.0056116)
Supplement: Table S3 — Primers and cycle conditions used in this study. (DOC) [file pone.0056116.s011.doc]

**Supplementary Table S3**

Primers and cycle conditions used in this study

| Gene | Access. no. | Primer | Cycle conditions |
| --- | --- | --- | --- |
| α-SMA | NM_007742 | For: 5’-GTTCAGTGGTGCCTCTGTCA-3’  Rev: 5’-ACTGGGACGACATGGAAAAG-3’ | Tm 60 ^o^C  No. of cycles: 40 |
| ColαI | NM_007392 | For: 5’-TAGGCCATTGTGTATGCAGC-3’  Rev: 5’-ACATGTTCAGCTTTGTGGACC-3’ |
| GFAP | NM_010277 | For: 5’-TTTCTCGGATCTGGAGGTTG-3’  Rev: 5’-AGATCGCCACCTACAGGAAA-3’ |
| p75 | NM_033217 | For: 5’-GGGGGTAGACCTTGTGATCC-3’  Rev: 5’-GTGTGCGAGGACACTGAGC-3’ |

* Abbreviations used are: IC, immunocytochemistry; WB, Western blot.
